# Involving People With Dementia in Research: Contributions, Barriers and Evaluation From a Meta‐Synthesis and Qualitative Interview Study

**DOI:** 10.1111/hex.70864

**Published:** 2026-09-07

**Authors:** Jörg Meisser, Susanne de Wolf‐Linder, Christina Ramsenthaler

**Affiliations:** ^1^ Department of Health Sciences Institute of Nursing, ZHAW Zurich University of Applied Sciences Winterthur Switzerland; ^2^ Wolfson Palliative Care Research Centre Allam Medical Building, Hull York Medical School, University of Hull Kingston‐upon‐Hull UK; ^3^ Cicely Saunders Institute of Palliative Care Research Policy and Rehabilitation, King's College London London UK

**Keywords:** dementia, evaluation, facilitators and barriers, patient and public involvement and engagement, qualitative interviews, qualitative meta‐synthesis

## Abstract

**Background:**

Patient and Public Involvement and Engagement (PPIE) can enhance the relevance, acceptability and quality of research; however, its meaningful implementation in dementia research remains insufficiently understood.

**Aim:**

(1) To identify methodological procedures for involving people with dementia (PwD) across the research lifecycle, and (2) to explore how members of a dementia PPIE group experience collaboration, contribution, and evaluation of their involvement.

**Methods:**

This mono‐method, multi‐strand qualitative study combined (1) a meta‐synthesis of qualitative primary studies reporting explicit PPIE with PwD and (2) semi‐structured interviews with members of a PPIE group. Both strands were analysed using qualitative thematic analysis. Findings were integrated through a crosswalk table and narrative synthesis.

**Results:**

The meta‐synthesis (33 studies) identified sequential, interrelated methodological conditions for PPIE with PwD across four themes: (1) access and recruitment, through trusted organisations and existing networks; (2) facilitation and support, using adapted communication, flexible pacing, practical aids, and sustained researcher facilitation; (3) forms of involvement, from consultation to co‐interviewing, co‐analysis, co‐authorship, and dissemination; and (4) evaluation of PPIE, which was inconsistently reported and rarely explicit about decision‐making impact. Qualitative interviews with 11 PPIE group members generated four experiential themes: (1) group‐related aspects (trust, appreciation, PwD representation), (2) structural conditions (organisation, preparation, continuity), (3) communication (clarity, accessibility, feedback), and (4) the handling and impact of contributions. Across strands, findings demonstrated strong convergence: methodological practices identified in the meta‐synthesis directly aligned with conditions under which participants experienced involvement as meaningful. Conversely, gaps in reporting and evaluation identified in the literature reflected participants’ concerns about limited visibility of impact.

**Conclusions:**

Effective PPIE with PwD requires sustained investment in relationship‐building, structured processes, accessible communication and continuous feedback to enable meaningful and inclusive collaboration. By integrating meta‐synthesis and experiential data, this study identified psychological safety, relational continuity and the visibility of influence as key in shaping meaningful involvement across research contexts.

**Patient or Public Contribution:**

Although people with dementia were not involved as research partners in study conception and design, the study sought to examine and incorporate lived‐experience perspectives through the included literature, qualitative interviews and participant validation workshop. This limitation is acknowledged and should be addressed through earlier involvement in future research.

## 1 Introduction

### 1.1 Patient and Public Involvement and Engagement (PPIE)

PPIE is an increasingly important element of international health research. It refers to the active involvement of patients, carers and the public in research; conducted *with* or *by* those affected rather than *about* or *for* them [1]. Incorporating experiential knowledge can enhance the relevance, quality and applicability of research findings [1, 2]. Evidence suggests PPIE helps identify practice‐relevant priorities, improve clarity of study materials, anticipate implementation barriers, and strengthen dissemination [2, 3].

PPIE is particularly well established in the United Kingdom (UK), supported by dedicated infrastructures and the UK Standards for Public Involvement, which emphasise inclusion, collaboration, support, communication, impact, and governance [4]. Evidence from the UK shows that PPIE can improve recruitment and the relevance and depth of data [2]. However, challenges persist, including unclear roles, tensions between professional and lay perspectives, and difficulties sustaining PPIE within research structures [2]. Similar approaches exist across Europe, but funding and institutional support remain uneven, with limited dedicated resources in many funding schemes [5]. Interest in PPIE has grown in Switzerland in recent years, supported by initiatives such as resources from the Swiss Clinical Trial Organisation [6], compensation mechanisms by the Swiss National Science Foundation [7], and the Swiss PPIE Network to strengthen coordination and knowledge exchange [8]. Despite this progress, PPIE remains less established than in countries with longer‐standing infrastructures, and empirical reflections on implementation are limited.

### 1.2 PPIE in Dementia Research

PPIE is widely considered essential in dementia research, with Alzheimer Europe describing meaningful involvement of people with dementia (PwD) as a ‘must‐have’ [9, 10]. However, PPIE in dementia research poses methodological, ethical, and practical challenges. Dementia, an umbrella term for progressive neurodegenerative conditions affecting memory, communication and executive functioning [11], can limit understanding, communication and sustained engagement in research [12, 13]. Additional challenges include the progressive nature, reliance on accompanying persons and logistical constraints such as transport and scheduling [3, 14, 15, 16]. Limited time and resources to build trusting relationships can further restrict meaningful involvement [14, 15], and sustaining participation over time remains difficult. Limited funding for remuneration, reimbursement and tailored support may further constrain active involvement by creating practical and financial barriers and restricting opportunities for PwD to contribute beyond advisory roles [1, 14]. These constraints may be compounded when the full costs of meaningful PPI, including staff time, coordination and ongoing support, are not adequately anticipated and costed in grant applications [17]. In the UK, such challenges are partly addressed through established organisations, such as the Alzheimer's Society or the UK Network of Dementia Voices, which provide coordination and support [13, 18].

Although PPIE is advocated across all research stages, evidence on how PwD contribute remains limited [16, 19]. Involvement most often takes advisory roles, with less frequent engagement in data analysis or dissemination [12, 14]. For example, PwD are typically involved as advisors and occasionally as co‐analysts, and only a minority of studies included in recent systematic reviews included contributions across multiple phases [12, 14]. However, opportunities exist throughout the research process, including co‐developing materials, supporting dissemination and contributing as co‐authors [2, 4, 5].

Evaluation of PPIE, particularly in dementia research, is also underdeveloped [20]. While implementation guidance exists, such as the Dementia Engagement and Empowerment Project (DEEP) guidelines [21], frameworks for systematic evaluation are limited. Tools like GRIPP2 [22] and approaches aligned with the UK Standards [23] have been proposed, but many studies provide only brief accounts, and formal evaluations are relatively rare [12, 14, 16, 19]. Moreover, reporting often lacks transparency on how PwD contributions influenced research [9, 13, 15], and issues such as ethics, power relations and structural conditions are inconsistently addressed [16, 24].

### 1.3 The Present Study

Against this background and in response to persisting gaps in empirical and evaluative evidence, this mono‐method, multistrand study combined a meta‐synthesis with a qualitative interview study to address a twofold aim: (1) To identify and analyse procedures and strategies that facilitate meaningful involvement of PwD in PPIE across different phases of the research cycle, and (2) to explore experiences of collaboration within an established PPIE group [25], from the perspective of PwD and other involved stakeholders, and to identify facilitating and hindering factors for subjectively beneficial PPIE practice.

## 2 Methods

This study adopts a mono‐method, multistrand qualitative design [26]. Objective (1), examining strategies for meaningful involvement, is addressed through a qualitative meta‐synthesis based on a systematic literature review [27]. Objective (2), exploring experiences of collaboration, is investigated via semi‐structured interviews based on the eSENIORS project at the Zurich University of Applied Sciences (ZHAW), one documented example of PPIE in dementia in Switzerland where PPIE groups contributed to developing an electronic tool to assess symptoms and needs among PwD [25]. Findings are integrated through triangulation using a crosswalk table and narrative synthesis [26]. The study is guided by a qualitative descriptive approach, aiming to represent participants’ perspectives close to the original meanings [28, 29, 30]. Accordingly, the study is grounded in a constructivist epistemological orientation [30]. Reporting follows the Preferred Reporting Items for Systematic Reviews (PRISMA) [31] in strand (1), and the Consolidated Criteria for Reporting Qualitative Research (COREQ) [32] in strand (2). No review protocol was registered prospectively. Completed checklists are available in Supporting Information S5: Appendix S5.

### 2.1 Part A. Meta‐Synthesis

#### 2.1.1 Search Strategy

A systematic literature review was conducted. Search terms covered three concepts: (1) dementia or mild cognitive impairment, (2) PPIE/participatory research, and (3) recruitment or involvement. These were expanded using synonyms and controlled vocabulary, adapted to each database, and combined with Boolean operators (see Table 1; full strategy in Supporting Information S1: Appendix S1).

**Table 1:** Basic search strategy.

Component	Search terms
Dementia	Dementia Cognitive dysfunction Mild cognitive impairment Alzheimer disease Memory disorders MCI AD
	AND
Patient and Public Involvement and Engagement	Patient and public involvement Patient and public involvement and engagement Service user involvement Patient participation Patient and public voice Study partner Participatory research Consumer participation Collaborative research Co‐research Citizen participation PPIE
	AND
Recruitment and involvement	Recruitment Participant engagement Inclusion strategies Involvement technique Participatory method Participant empowerment

#### 2.1.2 Eligibility Criteria

Eligibility criteria were defined using the Population–Phenomenon of Interest–Context (PICo) framework [33]. The population included people with any form of dementia, dementia‐like symptoms or mild cognitive impairment, with or without a formal diagnosis [34, 35]; mixed groups were eligible if at least one person with dementia participated. The phenomenon of interest was active involvement in at least one stage of the research process (PPIE). Studies were included if they explicitly described, reflected or evaluated PPIE in dementia research, using qualitative or mixed‐methods designs (with detailed PPIE reporting), and were published in English or German. Studies were excluded if PwD were only passive participants or had no influence on the research process, if they involved only unpaid carers or professionals in their PPIE groups, lacked explicit PPIE description, or used exclusively quantitative or non‐primary designs, as these were not expected to provide the interpretive detail required for this qualitative synthesis.

#### 2.1.3 Information Sources

The following electronic databases were searched (inception to 12/09/2025): PubMed, CINAHL, PsycINFO and LILACS (latter three via EbscoHost). No date limits were applied to capture earlier participatory approaches. A primary search string was developed for PubMed and adapted to other databases using Polyglot Search [36]. Validated qualitative research filters were applied where available [37, 38, 39]. Additional strategies included AI‐supported searches (Elicit) [40], citation tracking, and screening of reference lists and related articles. All records were managed in Zotero (v7.0.30) [41].

#### 2.1.4 Selection Process

Records were imported into Rayyan [42] for screening. After automatic and manual deduplication, titles and abstracts were screened against the eligibility criteria, followed by full‐text assessment. Screening was conducted by a single reviewer due to resource constraints, with full‐text decisions documented in line with PRISMA [31] and discussed within the author team where necessary.

#### 2.1.5 Quality Appraisal (Weight of Evidence [WoE])

Methodological quality and relevance were assessed using Gough's WoE [43] framework, rating (a) methodological quality, (b) methodological fit to the review question, and (c) relevance of context, population and focus as high, medium, or low. An overall WoE rating was assigned accordingly. Studies rated low were excluded, while higher‐rated studies were given greater interpretive weight. Ratings were conducted by a single reviewer.

#### 2.1.6 Data Extraction

Study characteristics (e.g., design, population, PPIE approach, key findings) were extracted into a structured, piloted Excel sheet by one reviewer. Relevant data on PPIE processes, contributions, facilitators, barriers and evaluation were imported into MAXQDA [44] for analysis.

#### 2.1.7 Synthesis Methods

The meta‐synthesis used a thematic qualitative analysis combining deductive and inductive analysis [45]. NIHR guidance [1] and the Alzheimer Europe position paper on PPIE [9] informed the operationalisation of the research questions and the development of analytical prompts to interrogate the data in the meta‐synthesis (see Supporting Information S2: Appendix S2). The deductive coding framework itself was based on the UK Standards for Public Involvement [4] and applied using defined coding rules. Following basic coding, new subcategories were developed inductively from the coded segments. These were refined through documented definitions and anchor examples. The full dataset was then recoded using the final framework, and categories were synthesised into higher‐order themes. WoE ratings informed interpretation, with greater weight given to higher‐quality studies. All analyses were conducted in MAXQDA (v24.9.1) [44] by a single reviewer.

### 2.2 Part B. Qualitative Interview Study

#### 2.2.1 Study Design

A qualitative descriptive design [28, 29] using semi‐structured interviews was used to explore participants’ experiences of collaboration within a PPIE group.

#### 2.2.2 Sample and Recruitment

Participants were recruited from the project's PPIE group using convenience sampling, guided by maximum variation to capture diverse perspectives [46]. The group comprised PwD, family carers, nursing professionals, and representatives of counselling and care services as well as non‐governmental organisations (NGOs) [25]. All members directly involved in research were invited via email and provided with study information. The target sample was 8–10 interviews (including 2–3 with PwD and 7–8 carers), with recruitment continuing until sufficient variation in perspectives was achieved (data saturation).

#### 2.2.3 Data Collection and Researcher Reflexivity

Data were collected through semi‐structured interviews using a topic guide (see Supporting Information S2: Appendix S2), focusing on experiences of collaboration, contributions, and facilitators and barriers to involvement. Interviews were conducted at participant‐chosen locations, lasted up to 90 min, and were audio‑recorded and transcribed verbatim. Field notes were taken and documented as analytic memos in MAXQDA [44].

Interviews were conducted by the first author, a registered nurse and, at the time of the interviews, project contact for the PPIE group for the past 2 months. The interviewer thus had a professional interest in PPIE. Direct contact with participants before the interviews was limited to email communication and brief interactions with four participants at a workshop. This prior contact supported rapport but may nevertheless have shaped interview interactions and the interpretation of accounts. Methodological rigour was ensured through piloting, reflexive practice, post‐interview reflections and regular supervision.

#### 2.2.4 Operationalisation and Analysis

The analytical framework was informed by the UK Standards for Public Involvement [4], which guided both the interview design and analysis (see Supporting Information S2: Appendix S2). Using a qualitative descriptive thematic approach, deductive and inductive coding were combined [46]. Analysis was conducted by a single researcher; to enhance transparency, coding decisions and category definitions as well as coding rules and anchor examples were documented in memos. Credibility was supported through member checking, with preliminary findings and the category system discussed and refined in a validation workshop with the PPIE group.

### 2.3 Ethical Considerations

The meta‐synthesis part used only published, anonymised data and did not require ethics approval [47]. The interview study followed the Declaration of Helsinki [48] and was approved by the ZHAW Research Ethics Committee (EA‐ZHAW‐2025‐STA‐007‐G). Participants received written information, provided informed consent, and re‑consented before the interviews.

As PwD may be vulnerable [10], additional safeguards were applied, including optional dyadic interviews, ongoing assessment of understanding and burden, and consideration of capacity in collaboration with family members to ensure informed participation [49]. Data were pseudonymised, securely stored, and accessible only to the research team.

## 3 Results

### 3.1 Part A. Meta‐Synthesis

**Figure 1:**
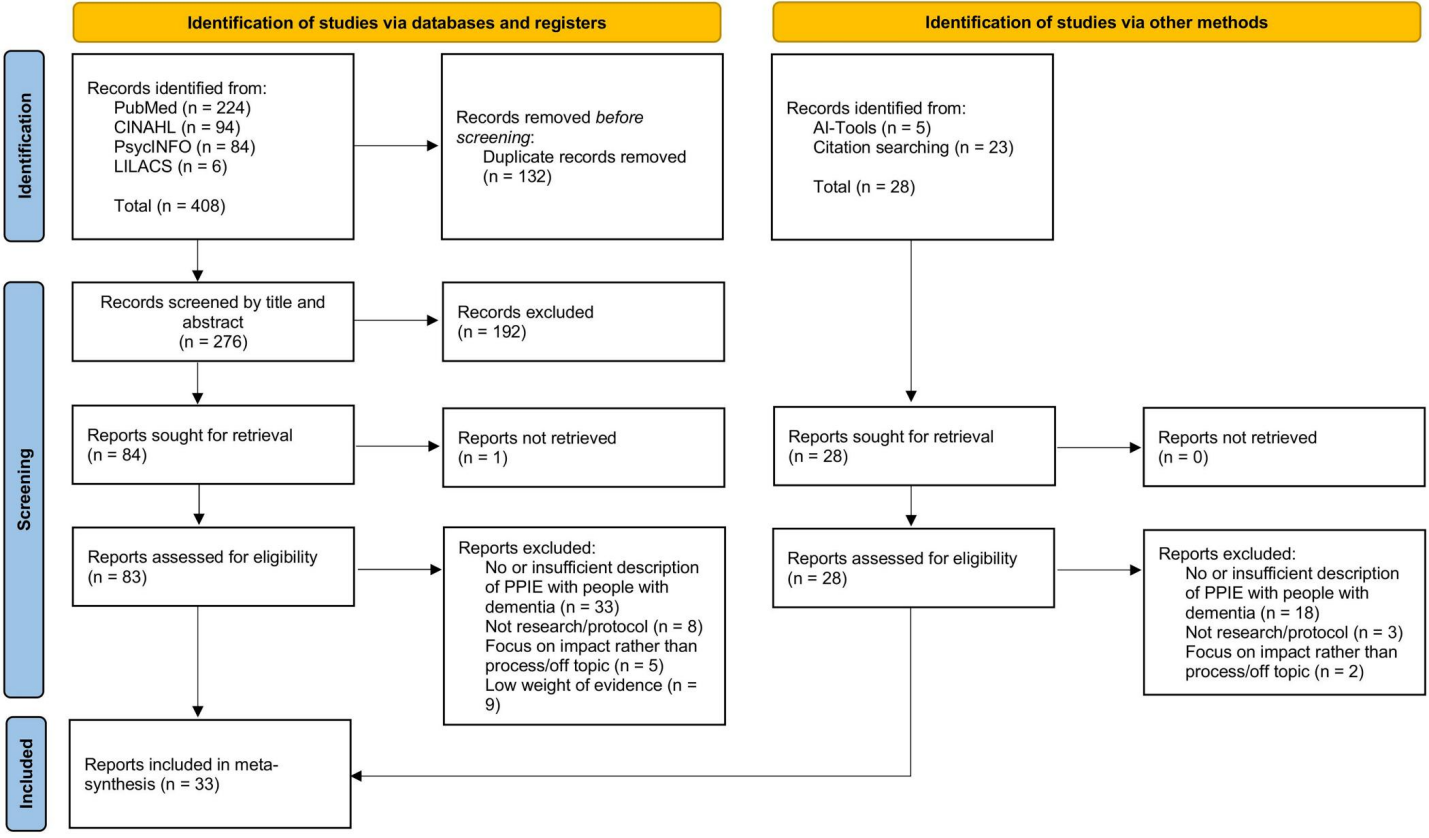
PRISMA flow chart.

A total of 408 records were identified, with 132 duplicates removed and 276 titles and abstracts screened. An additional 28 studies were identified through other methods. Following full‐text review and WoE appraisal, the latter resulting in removal of a further 9 studies, 33 studies were included in the meta‐synthesis (see Figure 1).

The synthesis primarily draws on studies with high WoE, with medium‐rated studies providing supplementary insights. The included studies were heterogeneous in their aims, designs, PPIE approaches and group composition, ranging from individual partnerships and small advisory groups to larger multi‐stakeholder groups. The description of PPIE activities, participant roles and group characteristics also varied considerably in depth and detail. An overview is presented in Table 2 (for detailed data see Supporting Information S3: Appendix S3). For quotations to illustrate themes, see Table 3.

**Table 2:** Characteristics of studies included in meta‐synthesis.

Study	Summary of study	PwD in the group (*n*)	Population/composition of the PPIE group	WoE rating (overall)
Beresford‐Dent et al. (2022) [49]	RCT with systematic embedding of PPIE; aim: to implement PPIE throughout the trial and to describe and evaluate it critically using GRIPP2.	Unclear	3–12: PPIE advisory group: PwD (various stages), family carers. Group size increased over time (2017–2019). One PwD was part of the Trial Steering Committee.	High
Bloska et al. (2024) [50]	Co‐designed survey of PPIE members in support of a multinational HOMESIDE RCT; aim: to assess the benefits, challenges, contributions and recommendations of PPIE‐aspects of the study.	4	32 members: 4 PwD, 15 family carers, 13 professionals across 5 countries.	Medium
Brooks et al. (2017) [51]	Qualitative descriptive mixed‐methods design (including review, focus groups and feasibility study); aim: to explore meaningful involvement of PwD as advisers and participants using Life Story Work as an example.	6	6 advisers with dementia supported by Innovations in Dementia; additionally, 25 PwD in focus groups; 39 PwD as participants in the feasibility study.	Medium
Capstick et al. (2021) [52]	Qualitative co‐creation in a PPIE setting; aim: to adapt PPIE so that PwD at different stages could take part in vignette co‐creation.	9	9 PwD of diverse age, gender and ethnicity, and 5 family carers.	High
Clarke et al. (2018) [53]	Participatory qualitative secondary data analysis in iterative workshops; aim: to involve PwD as co‐analysts and examine effects on interpretation and reflexivity.	20	34 co‐analysts: 20 PwD and 14 family carers.	High
Di Lorito et al. (2024) [54]	Multi‐stage co‐design/partnership process with underserved communities; aim: to report the PPIE partnership and co‐develop an information website for situations following a dementia diagnosis.	3	3 PwD and 4 former or current family carers.	High
Ditton et al. (2025) [55]	Reflexive PPIE development process within a doctoral project (collaboration with one PwD and a support person); aim: to summarise the PPIE approach, ethical dilemmas and effects on participation.	1	1 researcher, 1 PwD.	High
Donnelly et al. (2024) [56]	Hybrid PPIE event for instrument development; aim: to document collaboration with a Lewy body dementia advisory group in designing and pretesting a survey.	4	10 participants (4 PwD, 6 family carers).	Medium
Dooley (2022) [57]	Reflexive work in which the PPIE group analysed interaction data; aim: to reflect on roles, power balance and the contribution of PwD in interaction analysis.	12	12 PwD, 7 carers, 2 Alzheimer's Society group facilitators.	High
Dupuis et al. (2021) [58]	Participatory research approach with a described six‐step process; aim: to co‐develop a self‐management resource for PwD and care partners.	Unclear	PwD, family carers, representatives of the Alzheimer Society, First Link Coordinators, and healthcare professionals.	Medium
Fox et al. (2022) [59]	Agile app development as a co‐design process (continuous feedback, shared decision‐making); aim: to investigate the benefits of co‐designing an app and reflect on the process.	7	PwD and family carers: Workshop 1: 5 PwD, 2 carers; Workshop 2: 3 PwD, 3 carers; 1 PwD participated in both workshops.	Medium
Griffiths et al. (2024) [60]	Reflexive co‐production report (structured group reflection); aim: to present an example of a diverse PPIE group jointly developing a research grant application.	1	1 PwD, 4 family carers, 1 former family carer from culturally diverse backgrounds.	Medium
Hung et al. (2023) [61]	Methods/experience analysis across three projects (reflection discussions, notes, field notes, interviews; joint thematic analysis); aim: to examine telepresence as an innovative tool for participatory research during COVID‐19.	3	3 PwD and 3 family carers.	Medium
Kelly et al. (2025) [62]	Qualitative study using interpretative phenomenological analysis; aim: to describe and analyse the co‐writing of a song (including lyrics) and the experiences of PPIE contributors in the process.	3	PwD supported by carers.	High
Liddle et al. (2022) [63]	Meta‐reflection across three participatory technology design studies (team experiences); aim: to identify requirements, actions and barriers in building partnerships with PwD in technology research.	2	5 participants: 1 person with Alzheimer's disease and their partner, 1 person with frontotemporal dementia, 2 researchers.	High
Litherland et al. (2018) [64]	Reflexive group report; aim: to describe the work, methods, experiences and perceived impacts of an involvement group.	4+	9+: rolling membership, regularly including four PwD and five current or former family carers. Additional PwD and carers attended some meetings and discussed study materials.	High
Mann and Hung (2019) [65]	Action research (joint account by researcher and person with dementia); aim: to develop insights from the study as a shared perspective.	1	Person with dementia as co‐researcher.	High
Masoud et al. (2021) [66]	Qualitative interviews with thematic analysis (inductive‐deductive); aim: to describe experiences of members of a Stakeholder Advisory Council in the research process.	2	11 participants: 2 PwD, 4 family carers, 5 health or social care professionals with a connection to dementia.	Medium
Miah et al. (2020) [67] (Impact)	Mixed qualitative evaluation (monitoring forms and interviews); aim: to assess the impact of PPIE across all phases of the research cycle in a dementia research programme.	11	36 participants: 11 PwD, 12 family carers, 9 researchers and 4 project managers.	Medium
Miah et al. (2020) [68] (Training)	Evaluation study (acceptability scale and semi‐structured interviews); aim: to evaluate the acceptability and perceived effects of a training programme from the perspective of the PPIE group.	20	34 participants: 20 PwD, 14 family carers.	High
Middleton et al. (2025) [69]	Online co‐design; aim: to describe a participatory approach to developing the DELIGHT lifestyle intervention programme.	6	29 participants: PwD, family carers/relatives, various health professionals and community service providers.	Medium
Poland et al. (2019) [70]	Critical case analysis/conceptual reflection on PPIE including co‐research; aim: to critically evaluate the implementation of PPIE in a programme promoting independence in dementia.	Unclear	3 people with lived experience for the study; workshop groups in case 3.	Medium
Sakamoto et al. (2023) [71]	Participatory co‐design process plus interviews, analysed using reflexive thematic analysis; aim: to discuss and critically reflect on the experiences of people affected, carers and staff in the co‐design of an app.	1	9 participants: one person with dementia, family carers, and researchers in nursing, engineering, computer science and health design.	Medium
Sandoval et al. (2024) [72]	Modified Delphi approach as a virtual consensus conference; aim: to involve PwD in setting emergency medicine research priorities and identifying research gaps.	6	11 participants: four PwD, two family carers, two investigators, two project managers and one dementia advocate.	High
Smith et al. (2024) [73]	Reflexive practice report on establishing an advisory group within a doctoral project; aim: to reflect on opportunities, challenges, ethics and long‐term PPIE commitment when involving PwD.	3	5 participants: 3 PwD, 1 family carer, 1 health professional with a connection to dementia.	Medium
Snowball et al. (2022) [74]	Descriptive report; aim: to describe the activities, challenges and countermeasures of an advisory group within a programme‐level PPIE approach.	5	17 participants: 5 PwD, 12 current and former family carers.	High
Stevenson and Taylor (2019) [75]	Co‐research in qualitative analysis (co‐researchers derived meanings and identified/linked themes); aim: to report the process and effects of involving PwD in analysis.	4	4 PwD.	High
Tanner (2012) [76]	Participatory research design with co‐researchers (planning, interviews, interpretation); aim: to discuss the implications of involving PwD as co‐researchers.	3	3 PwD.	High
Thoft et al. (2020) [77]	Development of a participatory research model; aim: to provide collaborative support enabling PwD to develop their own research projects.	12	12 people with young‐onset dementia, divided into two groups.	High
Waite et al. (2019) [78]	Qualitative interview study with thematic analysis; aim: to identify facilitators and barriers to the co‐research involvement of PwD from different perspectives.	5	19 participants: 5 PwD, 6 researchers, 8 gatekeepers.	High
Warran et al. (2024) [79]	Co‐design/co‐writing through workshops and digital formats (including podcast); aim: to develop a shared research agenda on ‘dance and dementia’.	19	Workshop 1: 59 participants including 15 PwD, 10 family carers, 8 health professionals, 11 researchers, 9 artists, 6 cultural practitioners. Workshop 2: 23 participants including 4 PwD, 3 health professionals, 6 researchers, 4 artists, 4 cultural practitioners, 2 not specified.	High
Watson et al. (2023) [80]	Three‐phase participatory research project; aim: to develop, implement and evaluate a face‐to‐face support programme with, by and for people with lived experience of dementia.	40	97 participants: 40 PwD, 26 family carers, 31 health and social care professionals.	High
Wiersma et al. (2016) [81]	Ongoing discussion analysis with thematic analysis; aim: to analyse the debate on whether PwD should work in separate groups within research projects.	20	36 participants: 20 PwD, 13 family carers and 3 service providers.	High

Abbreviations: app, mobile application; COVID‐19, coronavirus disease 2019; DELIGHT, DEmentia Lifestyle Intervention for Getting Healthy Together; GRIPP2, Guidance for Reporting Involvement of Patients and the Public, Version 2; HOMESIDE, home‐based family caregiver‐delivered music and reading interventions for people living with dementia; PPIE, patient and public involvement and engagement; PwD, person/people with dementia; RCT, randomised controlled trial; WoE, weight of evidence rating.

**Table 3:** Quotations to illustrate themes in both parts of the study.

Part A. Meta‐synthesis			
Theme	Subtheme	Reference	Quote
A.1: Preconditions for involving PwD	A1.1: Relationship‐building and attitude	Mann and Hung (2019) [65]	“To develop trust, researchers need to stop making assumptions about what the person with dementia can contribute. […] For me, Jim, I wanted to be treated positively as a capable human being.” (Person with dementia)
		Waite et al. (2019) [78]	“Feeling safe is so important. I always had this safe feeling with her that if I got stuck I could just turn and ask her.” (Person with dementia)
	A1.2: Planning	Waite et al. (2019) [78]	“I could sit and discuss what people had said with you, maybe helping you to understand but if you gave me rows of figures to analyse or the text, forget it!” (Person with dementia)
		Wiersma et al. (2016) [81]	“You have to be very careful not to lay down hard and fast rules because you're dealing with two very highly complex relationships […]. And I think my preference would be – as with most of the rest of you – would be to exclude care partners. But I wouldn't make it a hard and fast rule. I think there may be lots of exceptions.“ (Person with dementia)
		Sakamoto et al. (2023) [71]	“Importantly, the virtual environment, while allowing the project to continue during pandemic restrictions, did prevent meaningful engagement with participants living with dementia. If able to conduct the co‐design workshops in‐person […] participants with dementia might have been able to express their thoughts about participating in the workshop sessions.” (Researcher)
	A1.3: Communication	Litherland et al. (2018) [64]	“[There's] a lot of support to us to get here for a start and to have good paperwork which we can understand. You know, you don't overwhelm us with stuff, the stuff that we receive is relevant, we can understand it.” (Person with dementia)
A.2: Enabling collaboration with people with dementia	A2.1: Support and training	Sandoval et al. (2025) [72]	“No matter what it was, they went out of their way to ensure that we got what we needed. They also were willing to have meetings with us beforehand, whatever took to bring us up to speed so we could be well prepared for the time when we had the conferences” (Person with dementia)
	A2.2: Tools and facilitation	Tanner (2012) [76]	“Although the initial preparation sessions were useful in providing a foundation of training, in practice the co‐researchers had little recall of this subsequently. A folder of simple summary information and the laminated ‘cue’ cards were invaluable in prompting memories of what had been covered.” (Researcher)
	A2.3: Narrative and creative approaches	Stevenson and Taylor (2019) [75]	“A series of prompts were presented to elicit views on what the group interpreted risk as meaning to a person with dementia (based on the participant responses), what they felt was interesting and how the responses connected together.” (Researcher)
**Part B. Interview study**	**Theme**	**Person speaking**	**Quote**
	B.1: Group‐related aspects	ID 10 (NGO)	“I think when there is a sense of ‘we’, then we have a goal, even if we come from different directions, even if we make different contributions, but if none of us is valued more highly or less highly, then I think that is an excellent way of working together.”
		ID 11 (Nursing professional)	“Yes, on the one hand the group gains new energy from every new member, but on the other hand, in terms of team spirit, I think that is less helpful.”
		ID 06 (Person with dementia)	“I was a bit afraid to contribute there. You notice that your own voice fades, that you become sad […]. So, feeling that deficit, in something that used to be what you were actually best at, at such an event then, well, I just don't know anymore.”
	B.2: Structural conditions	ID 09 (Nursing professional)	“As I remember it, there was always a clear question. A topic that we worked on and discussed together. And afterwards someone kind of summarised it. […] I thought that was good.”
		ID 04 (Family carer)	“And then at one point they said they would pass it on. But after that I didn't hear anything from them. Of course, it may also be that they do not publish at that frequency. Possibly there was also nothing to read. But after that I simply did not hear anything further.”
		ID 01 (Counselling professional)	“In the group, it might have been that I would have contributed. But online, I think I would have benefited more from the collaboration if I had been asked directly.”
	B.3: Communication	ID 06 (Person with dementia)	“At that moment, things were still quite clear for me. So at least I understood it then, and I didn't experience it as too professional, but rather as being able to say it in short key words, yes.”
		ID 02 (Care service provider)	“That happened relatively quickly for me. […] How it all fits together. That only really became clear to me the second or third time.”
	B.4: Handling and impact of contributions	ID 05 (Family carer)	“And perhaps it is the researchers, but perhaps it is someone else, like in your role, that when we have a group session, we bring in someone who has more of a facilitation role at the table. Simply so that the quieter ones do not get lost.”
		ID 08 (Family carer)	“Yes, those things appeared again afterwards in the minutes. Or I think they really try to implement what we give them as input.”

#### 3.1.1 Theme A.1: Preconditions for Involving PwD

Across studies, meaningful involvement of PwD emerged from the interplay of attitudes, relationships and structural conditions, rather than specific methods. Three domains were identified: relationship‐building and attitude, planning, and communication.

##### 3.1.1.1 Subtheme A1.1: Relationship‐Building and Attitude

Establishing trust between researchers and PwD is consistently identified as foundational for meaningful involvement in included studies [55, 60, 63, 64, 65, 67, 69, 76, 77, 78, 81]. This is supported by a respectful, non‐assumptive attitude that recognises PwD as capable contributors [65]. Relationship‐building begins at recruitment, where familiar settings and sufficient time facilitate informed engagement. Reported pathways include collaboration with organisations or networks [50, 53, 57, 58, 62, 64, 70, 75], existing PPIE groups [49, 51, 52, 55, 56, 59, 70, 76, 77], and personal contacts [50, 54, 69, 70, 73, 81].

Relationships are then actively maintained through ongoing contact, including informal exchanges and regular communication during quieter phases [49, 54, 55, 64, 69, 72, 76], fostering a safe and supportive environment for participation [78]. Clear yet flexible role definitions further support involvement: transparent expectations reduce uncertainty [54, 58, 66], while flexibility allows roles to adapt to changing interests, capacities and health over time [63, 73, 74].

##### 3.1.1.2 Subtheme A1.2: Planning

Early, systematic planning is a key prerequisite for meaningful involvement. Studies integrating PPIE at early stages report greater opportunities for co‐design and shared decision‐making, whereas later involvement is often constrained by pre‐defined structures [49, 50, 51, 63, 65, 77].

Planning involves assessing individual preferences, abilities and limits, with evidence that PwD can articulate their capacities in relation to specific tasks [58, 63, 68, 76, 77, 78]. At the organisational level, it includes flexible scheduling, accessible communication pathways and tailored support formats (e.g., one‐to‐one interactions or home visits) [49, 55, 58, 69, 77], as well as formal embedding of PPIE through clear agreements on roles, funding, support and reimbursement [49, 54, 70]. Some studies also describe dedicated PPIE coordinators as intermediaries [49, 64, 68].

Practical planning aspects include meeting length, group size and setting, typically involving sessions of 90–120 min, small to medium groups (2–12 participants), and adapted agendas [53, 54, 55, 60, 69, 75, 77]. Group composition is context dependent [81]. Accessible, familiar settings are preferred [49, 51, 53, 55, 64, 65, 77, 78], while online or hybrid formats may reduce logistical barriers but can hinder relationship‐building and require additional support [50, 54, 60, 69, 73, 74, 77].

Reimbursement and compensation are integral to planning, recognising participants’ expertise and covering participation‐related costs [50, 54, 55, 56, 59, 63, 64, 80].

##### 3.1.1.3 Subtheme A1.3: Communication

Accessible, responsive communication is a key prerequisite across all research phases. This includes using plain language, avoiding jargon, and providing information in multiple formats (e.g., visual materials, summaries and adapted texts) [49, 50, 51, 57, 60, 64, 66, 70]. Understanding is supported through ongoing clarification, opportunities for questions and repetition of key concepts [50, 60, 64]. Regular updates and reliable communication channels further sustain engagement and continuity [54, 63, 67, 69, 74].

#### 3.1.2 Theme A.2: Enabling Collaboration With PwD

Beyond foundational conditions, studies describe how meaningful involvement is enabled in practice through support, facilitation and methodological adaptation.

##### 3.1.2.1 Subtheme A2.1: Support and Training

Support spans organisational, technical and emotional domains, including transport assistance, accompaniment, preparation, and help with digital participation, alongside relational support [55, 63, 72, 74, 75]. Training is less frequently reported but, where present, is brief, task‐specific and practice‐oriented, often supported by visual materials [68, 77].

##### 3.1.2.2 Subtheme A2.2: Tools and Facilitation

Tools such as visual aids, summaries and cue cards help structure participation and reduce cognitive demands, also serving as memory aids for complex tasks [49, 56, 62, 76, 77, 80]. Facilitation is crucial for maintaining engagement, using strategies such as framing discussions, inviting contributions, pausing, repeating information and checking understanding [53, 54, 55, 57, 60, 66, 69, 79].

##### 3.1.2.3 Subtheme A2.3: Narrative and Creative Approaches

Narrative and creative methods enable expression beyond text‐based formats, including storyboards, graphic vignettes, visual prompts, videos, quotations, and activities such as songwriting or drawing [52, 53, 56, 57, 62, 75, 77]. To support authentic representation, studies highlight strategies to reduce power imbalances, such as participant validation, protected spaces and reflexive, non‐directive communication [59, 62, 66, 70, 75, 81].

#### 3.1.3 Theme A.3: Forms of Involvement

Across the included studies, PwD contributed in diverse roles across all phases of the research process (study planning to dissemination), ranging from one‐time consultation to sustained involvement as co‐researchers (for details see Supporting Information S4: Appendix S4).

#### 3.1.4 Theme A.4: Evaluation of PPIE Activities

Evaluation was reported using both informal and formal approaches. Informal methods included ongoing reflection through debriefings, group discussions and iterative feedback [55, 60, 63, 72, 77]. Formal approaches comprised qualitative interviews, focus groups, structured evaluation events and questionnaires assessing benefits, challenges and impact [50, 64, 66, 68, 72, 74, 75]. Some studies used reporting frameworks such as GRIPP2 and monitoring tools (e.g., activity logs), while a few applied creative methods such as songwriting [50, 56, 62, 68].

### 3.2 Part B. Interview Study

Ten interviews (9 individual, 1 dyadic) were conducted with 11 participants, including one PwD, family carers, nursing professionals, and representatives of care and counselling services; some held overlapping roles. Interviews took place at participant‐chosen locations. Prior contact with the interviewer was minimal, limited to email introduction and brief interaction with four participants at a workshop.

Analysis identified three main themes shaping collaboration: (1) group‐related aspects, (2) structural conditions, and (3) communication, each encompassing facilitating and hindering factors. A fourth theme addressed experiences of contribution and impact. Data suggested saturation for facilitating aspects, while hindering experiences remained more heterogeneous.

#### 3.2.1 Theme B.1: Group‐Related Aspects

Group dynamics were experienced as both enabling and constraining. An open, respectful and trusting atmosphere—valuing diverse perspectives and shared connections to dementia—supported meaningful contributions, with appreciation, particularly towards PwD, seen as essential.

At the same time, changing group composition had mixed effects: new members introduced fresh perspectives but could disrupt continuity and trust‐building. Limited presence of PwD was perceived as constraining, potentially reinforcing vulnerability and hesitation to contribute. Differences in energy and capacities also posed ongoing challenges requiring sensitive facilitation.

The research team was perceived as enabling when acting consistently and fostering an appreciative, inclusive stance, contributing to psychological safety within the group.

#### 3.2.2 Theme B.2: Structural Conditions

Structural factors were central to participation. Clear organisation, preparation and facilitation, through structured meetings, clear agendas and written documentation, supported engagement and conveyed the seriousness of the work. Support mechanisms, including designated contacts and targeted attention to PwD and carers, enhanced safety and inclusion. Group size involved a trade‐off between active participation (smaller groups) and diversity of perspectives (larger groups).

Constraints arose where support was limited, such as insufficient follow‐up communication, weak inclusion of online participants, or scheduling conflicts. Short‐notice meetings and competing commitments further hindered participation.

Hybrid formats were valued for flexibility, but face‐to‐face interaction was preferred. Online and hybrid settings limited informal exchange, non‐verbal communication and spontaneity, sometimes reducing a sense of belonging. Inadequate facilitation of online participants further constrained engagement, particularly for more reserved individuals.

#### 3.2.3 Theme B.3: Communication

Communication was a cross‐cutting factor influencing collaboration. It facilitated involvement when clear, transparent and well structured, with concise, accessible information on aims, procedures and expectations, supported by preparatory materials and summaries. Active facilitation, such as asking follow‐up questions, summarising and revisiting inputs, and the use of plain language were considered particularly helpful.

Conversely, unclear or insufficiently transparent communication hindered collaboration, especially at the outset. Participants reported difficulties understanding links between project components, unclear task allocation and overly technical language. A lack of connection between individual meetings and overall project goals further reduced perceived relevance.

#### 3.2.4 Theme B.4: Handling and Impact of Contributions

Participants evaluated collaboration primarily by how their contributions were acknowledged and incorporated. Meaningful involvement depended on whether inputs were visibly taken up, reflected and traceable in subsequent steps. Workshops were often experienced as collaborative processes in which ideas evolved through discussion; although expectations were not always clear initially, their clarification during the process was generally accepted.

Facilitation played a key role in supporting inclusion, particularly through prompting quieter participants, summarising discussions and integrating perspectives. Written documentation and explicit feedback were important for recognising contributions. Perceived impact became evident when inputs were reflected in meeting minutes and outputs, strengthening trust and reinforcing the sense of meaningful participation. Many participants also described the process as mutually beneficial for both researchers and participants.

### 3.3 Summary and Integration of Key Findings

Four interrelated themes relevant to meaningful PPIE with PwD were identified in the meta‐synthesis: preconditions for involvement, enabling collaboration, forms of involvement and evaluation of PPIE activities. In the interview study, four experiential themes were identified: group‐related aspects, structural conditions, communication, and the handling and impact of contributions.

A systematic integration of both parts is shown in Table 4, cross‑walk analysis, identifying where themes converge, complement or contradict each other. Across both strands, there is strong convergence on the central role of relationship‑building as the foundation for trust and an open, appreciative atmosphere, which together enable psychological safety. However, findings also highlight that such safety is dynamic and must be actively sustained, particularly where group composition changes or PwD are underrepresented. Similarly, both datasets emphasise the importance of role clarity, while demonstrating that it is not static but requires ongoing negotiation; this is closely linked to the need for flexible roles that can accommodate changing capacities and heterogeneous needs. Organisational and structural conditions show consistent alignment, identifying the research team as a key enabler of participation through coordination, support and the provision of clear structures. Complementary insights indicate that early embedding and visible professionalism not only facilitate co‑design but also signal legitimacy and recognition to participants, reinforced by designated coordination roles. Managing heterogeneity emerges as a core challenge, requiring active calibration of group composition, size and format rather than standardised approaches. Communication functions as a cross‑cutting mechanism, with accessible formats, plain language and continuous feedback enabling both understanding and sustained engagement, while gaps in feedback undermine perceived relevance. Facilitation and support further operate as critical mechanisms translating inclusive intentions into practice, although their effectiveness depends on context‑sensitive adaptation, particularly in online settings. Digital formats illustrate a key tension between increased accessibility and reduced relational quality. Finally, meaningful involvement is closely tied to the visibility of contributions, with authenticity realised when inputs are traceable in decision‑making processes. Overall, the integration highlights that meaningful PPIE is shaped by the interplay of process quality and structural conditions, requiring continuous adaptation and reflexivity.

**Table 4:** Cross‐walk‐table.

Meta‐synthesis: theme/core aspect	Interview study: link/finding	Relationship	Implication
Relationship‐building and underlying attitude (trust, open attitude, ongoing relationship work)	Group‐related aspects: open/friendly atmosphere, appreciation as a prerequisite for contributing	Congruence	Psychological safety
Relationship work starting at recruitment (familiar settings, time to get to know one another)	Group‐related aspects: hesitation and burden when only few PwD were present, emotional impact/experience of deficit	Complementarity	Vulnerability within the setting
Clarity of roles, responsibilities and expectations (agreements, introduction, where appropriate in writing)	Communication/structure: initial lack of clarity regarding purpose/aim, wish for clearer task allocation	Congruence	Role clarity from the outset
Role flexibility (abilities/capacity may change)	Group‐related aspects: cognitive/personal differences, differing energy levels as a risk	Congruence + complementarity	Flexible involvement that responds to individual differences
Early embedding (more resources/opportunities for co‐design)	Structure: good preparation/professionalism experienced as facilitating; structure signals seriousness	Complementarity	Embed early and convey professionalism
Clarification of skills, preferences and limits	Group‐related aspects: cognitive/personal differences, differing energy levels as a risk	Congruence + complementarity	Managing heterogeneity
Organisation (scheduling, suitable communication channels, individual contact/transport where needed)	Structure: scheduling/short‐notice appointments as a barrier, team support as facilitating	Congruence	Research team as supporter
Formal embedding (roles/processes/funding, PPIE leads as link persons)	Structure: designated contact person viewed positively; online participants benefited from active support by a team member	Congruence	Coordination and bridging roles are important
Time/place/group/structure (90–120 min, familiar location, small to medium‐sized groups, breaks, reduced agenda)	Structure: smaller groups facilitate contribution; larger groups provide more perspectives; workshop structure is important	Congruence	Dosage, group size and format
Online/hybrid formats (barriers vs reach, additional support required)	Structure: online/hybrid formats hinder non‐verbal cues, informal conversations and sense of belonging; lack of active prompting online	Tension/complementarity	Advantages and disadvantages of hybrid/online settings
Reimbursement/cost coverage (recognition, expenses)	Not addressed in the interviews	—	—
Comprehensible presentation (visualisations, short summaries, multiple formats, large font)	Communication: information experienced as suitable/appropriate; preparation and follow‐up (tasks/minutes) seen as helpful	Congruence	Practical comprehensibility
Avoidance of jargon/use of plain language, ensuring understanding	Communication: technical terms in tasks were a barrier; misunderstandings were possible	Congruence	Use plain language
Reliable communication structures + one‐to‐one contact	Structure/communication: feedback sometimes absent, experienced as hindering	Congruence	Continuous feedback is important
Support (organisational/technical/emotional)	Structure: team support experienced as facilitating; online participants benefited from active support/representation	Congruence	Support as a mechanism
Training (brief, modular, close to tasks)	Not addressed in the interviews	—	—
Tools (memory aids, cards, interview cue cards)	Not addressed in the interviews	—	—
Facilitation (framing, targeted invitations, follow‐up questions, repetition)	Evaluation of input/structure: active facilitation strengthens belonging; lack of active prompting online hinders contribution	Congruence + tension	Facilitation as key, but demanding
Creative methods (vignettes, images/prompts, songwriting, drawing)	Not addressed in the interviews	—	—
Authenticity/power asymmetries (validation, settings with only PwD, non‐suggestive communication, flexible methods)	Evaluation of input: it is important that contributions are visibly taken up and remain recognisable (trust/impact)	Complementarity	Make influence visible
Involvement across research phases (from pre‐trial to dissemination; table by phases)	Interview study focused on conditions for collaboration (group/structure/communication), less on phases	Complementarity	Process logic vs. phase logic
Informal and formal evaluation (reflection/debriefing, interviews; questionnaires, GRIPP2)	The qualitative interview study itself represents an evaluation process	Congruence	Suitable form of evaluation

**Figure 2:**
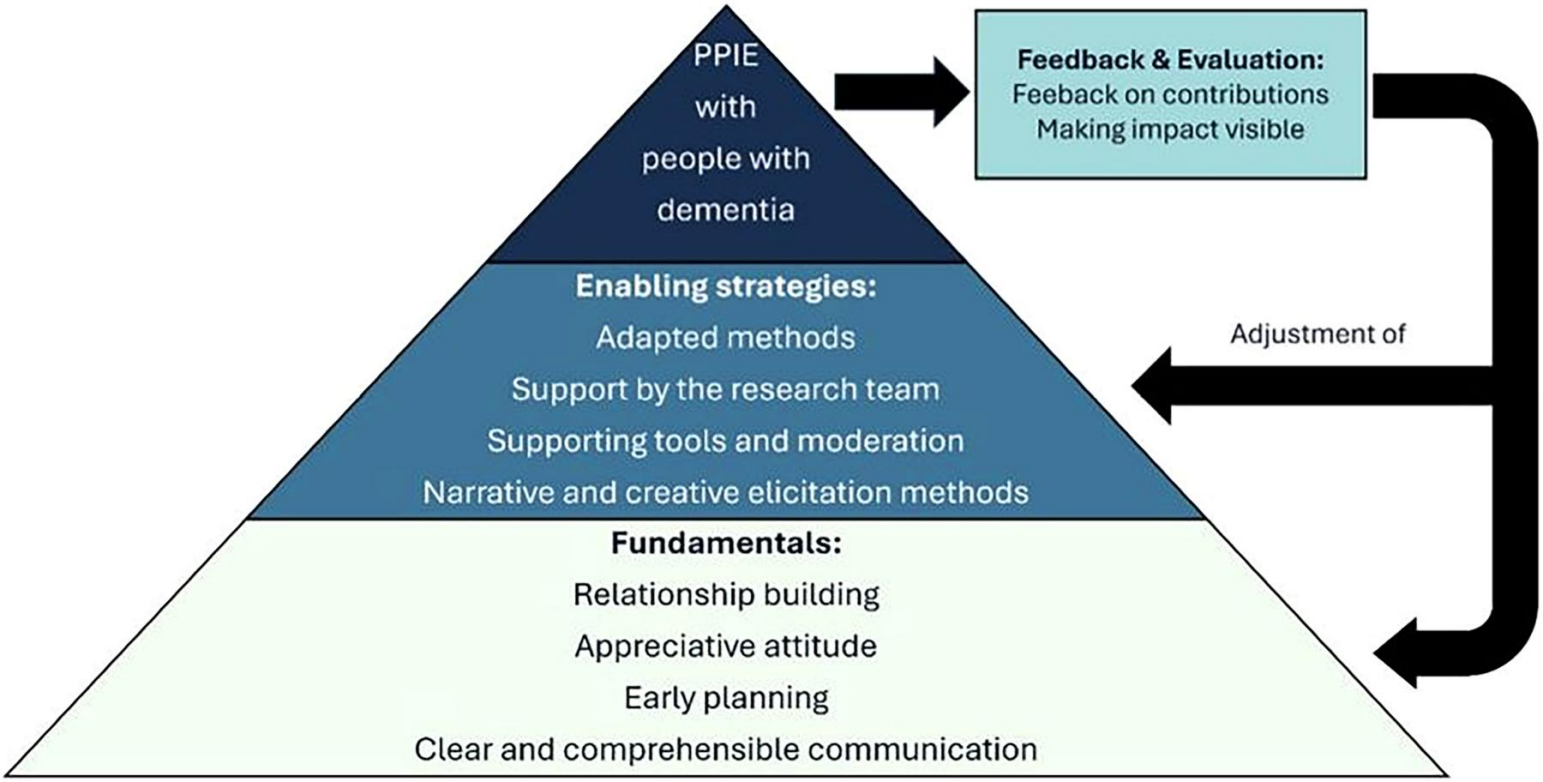
Integrative model of achieving meaningful involvement for PwD in dementia research.

The integrated findings indicate that meaningful PPIE is less defined by specific methods than by how collaboration is organised, communicated and enacted in relational and structural terms. Figure 2 presents the resulting integrative model, highlighting the foundational role of relational, organisational and communicative conditions in enabling meaningful PPIE in dementia research.

## 4 Discussion

### 4.1 Interpretation in the Context of the Literature

This mono‐method, multistrand study provided a comprehensive analysis of meaningful involvement of PwD in research by integrating evidence from a meta‑synthesis with findings from a qualitative interview study, giving a practice‑oriented insight into how meaningful involvement is established, enacted and experienced.

In line with NIHR and DEEP guidance [1, 21] and prior reviews emphasising the importance of time and relationship work in dementia PPIE [15, 16], this study confirms that meaningful involvement is fundamentally relational and processual. It extends these existing reviews by demonstrating how relationship‐building, planning and communication operate not as isolated factors but as interdependent and cumulative conditions underpinning all subsequent involvement activities. Similarly, while existing guidance highlights the need for role clarity and transparency [1, 13], our findings show that these are not stable achievements but require continuous negotiation, particularly in early stages, thereby refining current recommendations towards a more dynamic understanding of role development.

In addition, while previous reviews indicate that PwD are most often involved in advisory roles [12, 14], the included studies demonstrate that contributions can extend across the research cycle. Our integrated findings suggest that extending involvement beyond consultation depends on early planning, flexible roles, task‐specific preparation and sustained facilitation.

The study is also consistent with previous work in identifying support and continuity as central facilitators [1, 13, 21], yet adds nuance by showing how structured processes, immediate assistance and designated contact persons contribute not only to practical feasibility but also to trust, reliability and recognition. This complements existing frameworks by specifying relational continuity as a key mechanism of effective PPIE. Findings on hybrid formats align with literature describing both opportunities and challenges of digital inclusion [18], but further suggest that limitations arise less from the format itself than from unequal facilitation and support, highlighting the need for active design of equivalent participation.

The prominence of visual aids, structured summaries and narrative or creative approaches further indicates that accessibility can be achieved through different venues. This is consistent with existing guidance recommending dementia‐friendly materials, advance preparation, explanations and appropriate training [10, 21], and with previous reviews describing visualisation, familiar objects, storytelling and other adapted approaches to support involvement [3, 16, 82]. However, dementia‐adapted and creative methods remain inconsistently described and, in some areas of PPIE, appear to be used only rarely [19]. This study shows how visual aids, summaries and cue cards may function as cognitive scaffolds for orientation, memory and engagement with complex tasks, while narrative and creative approaches can provide alternative ways of expressing experience and contributing to interpretation beyond text‐based or fast‐paced discussion. Importantly, this study extends the evidence base by foregrounding group composition and psychosocial dynamics. These areas have been less explicitly addressed in prior research. Experiences of vulnerability when few PwD were present illustrate that inclusion is not solely structural but also relational and emotional. Finally, addressing a well‐documented gap around evaluation of PPIE activities [12, 14, 15, 19], our findings provide empirical insight into how impact is experienced: not only through formal outcomes but through the continuous visibility and traceability of contributions.

### 4.2 Implications for Policy and Practice

The integrated findings suggest several implications for dementia research involving PPIE. First, PPIE should be embedded early and structurally, with the form and extent of involvement tailored pragmatically to the project context, contributor preferences and capacities. This requires adequate resources for coordination, reimbursement and support and/or training for both the research team and people with lived experience to enable genuine co‐design. Second, relationship‐building should be treated as a core methodological component, supported by team continuity, informal interactions and clear points of contact. Third, dementia‐friendly communication, i.e., using plain language, visual materials, repetition and active facilitation, should be applied consistently. Fourth, online and hybrid formats require tailored support and facilitation to avoid exclusion. Finally, evaluation should be integrated throughout, with structured feedback linking contributions to decisions and documenting impact, supported by tools such as GRIPP2 [22] alongside process‐oriented approaches.

### 4.3 Strengths, Limitations and Future Research

This study combines a systematic meta‐synthesis with primary qualitative data, providing both breadth and contextual depth. However, several limitations should be noted.

In the meta‐synthesis, screening, WoE appraisal and analysis were undertaken by a single reviewer. This represents a substantial methodological limitation that may have influenced study selection, appraisal, and interpretive processes as eligibility decisions, judgements of study relevance and quality, and the development of categories and themes were not independently checked. However, decisions were documented throughout the review process and regularly discussed with the co‐authors, providing opportunities for critical reflection and refinement of interpretations. Furthermore, no review protocol was registered. The review had already commenced before prospective protocol registration was considered. We recognise that the absence of a registered protocol reduces methodological transparency and may increase the risk of post hoc methodological decisions. In the interview study, perspectives of PwD were limited to one participant, reducing transferability. Analysis was also conducted by a single researcher and was therefore not independently corroborated; however, transparency was supported through documentation of analytic decisions, and preliminary findings were discussed and refined with the PPIE group in a validation workshop.

Although the interview study drew on an established PPIE group, group members were not involved in setting the study aims or designing the study. The study scope and key methodological decisions had already been established before their involvement. Consequently, lived‐experience perspectives could not shape foundational decisions. This means that the study did not enact the principles of early and shared involvement that it examines and advocates. The validation workshop enabled feedback on preliminary interpretations and refinement of findings, but did not constitute co‐design or shared decision‐making regarding the study aims, scope or methods. Future research should involve people with lived experience before key decisions are made and report transparently the scope and influence of their involvement.

Future research should expand direct involvement of PwD as research partners, including tailored roles across research phases, develop robust approaches to evaluating PPIE processes and impact, and explore how digital and hybrid formats can support equitable participation.

## 5 Conclusion

Meaningful involvement of PwD in research is grounded in relationship‑building, structured planning and accessible communication, supported by ongoing facilitation and adaptive support. Hybrid and digital formats pose additional challenges and require deliberate design. Crucially, making the impact of contributions visible through transparent feedback processes enhances trust and the perceived value of participation. Future efforts should focus on strengthening evaluation practices, clarifying roles within PPIE, and supporting inclusive participation across diverse contexts.

## Author Contributions

**Jörg Meisser:** conceptualisation, data curation, formal analysis, investigation, methodology, software, validation, visualisation, writing – original draft preparation, writing – review and editing. **Susanne de Wolf‐Linder:** conceptualisation, methodology, project administration, supervision, validation, writing – original draft preparation, writing – review and editing. **Christina Ramsenthaler:** conceptualisation, formal analysis, methodology, project administration, supervision, validation, writing – original draft preparation, writing – review and editing.

## Funding

The authors have nothing to report.

## Ethics Statement

The meta‐synthesis component of the study was based exclusively on previously published, anonymised data and therefore did not require ethics committee approval. The interview study was conducted in accordance with the principles of the Declaration of Helsinki and Good Clinical Practice (GCP) and was approved by the Research Ethics Committee of the Zurich University of Applied Sciences (Ref No.: EA‐ZHAW‐2025‐STA‐007‐G).

## Consent

All interview participants received written study information and provided written informed consent prior to participation; verbal re‑consent was obtained immediately before the interviews. For participants with dementia, specific safeguards were applied, including dyadic interviews where appropriate and continuous assessment of understanding, willingness to participate and potential burden during the interviews.

## Conflicts of Interest

The authors declare no conflicts of interest.

## Supporting information


**Appendix S1:** Full search strategy.

**Appendix S2:** Operationalisation and topic guide.

**Appendix S3:** Detailed data extraction table and weight‐of‐evidence ratings.

**Appendix S4:** Methods for meaningful involvement along the research cycle (additional results).

**Appendix S5:** Completed PRISMA and COREQ checklists.

## Data Availability

The data that support the findings of this study are available on request from the corresponding author. The data are not publicly available due to privacy or ethical restrictions. The qualitative interview data generated and analysed during this study contain potentially identifiable information. In accordance with the informed consent provided by participants and the conditions of the ethics approval, these data cannot be shared publicly. Limited access may be considered by the corresponding author where this is consistent with ethical requirements. Data used for the meta‑synthesis are based exclusively on previously published studies and are available within this article and through the original publications referenced.
