# Supplementary material for: Involving People With Dementia in Research: Contributions, Barriers and Evaluation From a Meta‐Synthesis and Qualitative Interview Study
Source: Health Expect. 2026 Sep 7;29(5):e70864. doi: 10.1111/hex.70864 (PMC13548409; doi:10.1111/hex.70864)
Supplement: Supplementary file 1 [file HEX-29-e70864-s001.docx]

**Data extraction table**

| **First author, year** | **Journal** | **Aim** | **Design / Method** | **Population (PPIE-Group)** | **# of PmD in PPIE-Group** | **Eligible for Meta-Synthesis?** |
| --- | --- | --- | --- | --- | --- | --- |
| Beresford-Dent, 2022 [1] | Dementia | To embed PPIE throughout a randomised controlled trial of a psychosocial intervention called ‘Journeying through Dementia’ | Use of the GRIPP2 checklist to describe and critically appraise the approaches to PPIE within the study | PPIE advisory group: PwD (various stages, some newly diagnosed, some more advanced), family supporters (often spouses/relatives). Group size grew from 3 to 12 members over time (2017–2019).  One PPI member in Trial Steering Committee (dementia diagnosis). | Unclear | Yes |
| Bielsten, 2018 [2] | Qualitative Health Research | To identify relevant content for a self-management guide for couples where one partner has a diagnosis of dementia, combining previous research with lived experience. | Methodology: Alfred Schütz's framework of first- and second-order constructs. Methods in 3 Phases:  Phase 1: Literature search Phase 2: 10 (first and follow-up) semi-structured, dyadic interviews with couples. Phase 3: Consultation meetings with expert groups of PwD and carers in Sweden and the UK. | 5 Swedish couples (n=10; mixed gender, aged 67–77, Alzheimer’s diagnoses)  Consultation groups: 40 people with dementia and carers in Sweden, 6 couples in UK. | 5 + unclear | No |
| Bloska, 2024 [3] | Research Involvement and Engagement | To capture experiences of participant and public involvement across five countries in the HOMESIDE RCT, exploring benefits, challenges, and contributions. | Co-designed survey of PPI-members covering 4 topics: expectations, perceived contributions, benefits & challenges and recommendations for future research.  Following survey report checklist and GRIPP2 checklist. | 32 members: 4 people with dementia (12.5%), 15 family caregivers (46.9%), 13 professionals (40.6%) across 5 countries. | 4 | Yes |
| Bosco, 2019 [4] | Archives of Gerontology and Geriatrics | To develop an integrated dementia care model that promotes personhood through co-production, drawing on systematic reviews, policy analysis, and PPI input. | Meta-ethnographic approach to synthesise literature on personhood and dignity in dementia care using electronic databases and relevant policy documents. Members of PPI-group were consulted throughout. | Established group in Nottingham; included PwD and carers, predominantly white British, aged 60–70 | Unclear | No |
| Brooks, 2017 [5] | Sheffield University Research Archive | To explore how people with dementia can be meaningfully involved in research as advisers and participants, using life story work as the case study. | Qualitative descriptive and mixed-methods approach (systematic review, focus groups, feasibility study with QoL outcome measures) | 6 advisers with dementia supported by Innovations in Dementia; additional 25 people with dementia in focus groups; 39 participants with dementia in the feasibility study | 6 | Yes |
| Capstick, 2021 [6] | Health Expectations | To adapt the PPIE process so that people living with dementia across a wide range of cognitive abilities can meaningfully participate in the co-creation of narrative and graphic vignettes of lived experience. | Qualitative co-creation study using narrative elicitation, photo-elicitation, informal conversation, and observation within a PPIE framework; iterative development of text and graphic vignettes. | 9 people living with dementia (diverse in age, gender, and ethnicity) and 5 family members; recruited through a university PPIE panel and a community resource centre. | 9 | Yes |
| Clarke, 2018 [7] | Qualitative Health Research | To explore ways of involving people living with dementia as co-analysts in qualitative secondary data analysis, examining how participatory methods can reshape data interpretation, researcher reflexivity, and authority in knowledge production. | Participatory qualitative secondary data analysis; iterative workshop-based design using two theoretical frameworks: Douglas’s Cultural Theory of Risk and Tronto’s Ethic of Care; cycles of presenting, interpreting, and reinterpreting data. | 34 co-analysts (people with dementia and family care partners) from 4 pre-existing Alzheimer’s Society and Alzheimer Scotland groups in England and Scotland | 20 | Yes |
| Clarke, 2020 [8] | Dementia | To perform secondary data analysis with PwD on how social relationships and care interactions shape inclusion and exclusion. | Same as above | Same as above | - (same as above) | No |
| de Wolf-Linder, 2024 [9] | Frontiers in Dementia | To describe the methods, format, and lessons learned in co- creating and co-producing a dissemination strategy for a research project focused on establishing patient-centred outcome measures into routine palliative community care for PwD and their informal carers. | Participatory, hybrid-format workshop was conducted to co-create the dissemination strategy with a PPIE group. The workshop was followed up with a survey to consolidate the dissemination strategy. Workshop minutes and survey responses were analysed using qualitative thematic analysis. | 2 PwD, 10 family members, 22 nurses, 9 NGO (4 double roles) | 2 | No |
| Di Lorito, 2024 [10] | Health Expectations | To report on a PPIE partnership between academics and members of the public from underserved communities to co‐design ‘Forward with Dementia—Social Care’, a resource and information website supporting people receiving a dementia diagnosis. | Four stages: (1) identifying communities that have been under‐represented from PPIE in dementia research; (2) recruiting PPIE partners from these communities;(3) supporting PPIE partners to become confident to undertake their research roles; (4) undertaking research co‐design activities in an equitable fashion | 4 former or current carers, 3 PwD | 3 | Yes |
| Ditton, 2025 [11] | Research Involvement and Engagement | To summarise the PPIE process taken to develop a piece of doctoral research. | This project involved collaboration with an individual living with dementia who was previously unfamiliar with research practices, and their friend. They combined a practical PPIE framework with an appreciative research approach to reflect on concepts such as ethical dilemmas and how these impact on participation. | 2 | 1 | Yes |
| Donnelly, 2024 [12] | Frontiers in Dementia | To report on the outcomes of collaboration with a Lewy body dementia research advisory group during the design phase of a survey. | Conducting the stages of end-user engagement and pretesting together during a public involvement event. A hybrid approach involving a focus group with breakout interviews was employed. Feedback from contributors informed the evolution of the survey instrument. | 10 (4 PwD, 6 carers, one of which is co-author) | 4 | Yes |
| Dooley, 2022 [13] | International Journal of Social Research Methodology | To reflect the involvement of PwD in interaction analysis and balance of power between researchers and PPI contributors. | Data sessions of 4 recordings were analysed by PPIE-group | 12 PwD, 7 carers, 2 Alzheimer's Society group facilitators | 12 | Yes |
| Dupuis, 2021 [14] | Dementia | To describe use of participatory action research (PAR) to developing a self-management resource for PwD and care partners. | Description of six-step process from establishment of PAR-Team to final resource creation. | PwD, care partners, Alzheimer Society representatives, First Link Coordinators, actors in healthcare systems | Unclear | Yes |
| Fox, 2022 [15] | JMIR Mhealth Uhealth | To explore benefits of co-design of an app and reflect on the process. | App was developed using Agile methodology based on 3 principles: end-user representation, equal decision-making power and continuous user consultation, testing & feedback | Mix of PwD and carers: Workshop 1: 5 PwD, 2 carers Workshop 2: 3 PwD, 3 carers 1 PwD attended both workshops. | 7 | Yes |
| Griffiths, 2024 [16] | Dementia | To provide an example of a diverse lived experience group co-producing a dementia research funding application. | Reflection of experiences based on a co-produced set of questions. Reflections were then structured by the group. | 1 PwD, 4 carers, 1 former carer, culturally diverse. | 1 | Yes |
| Harrington, 2022 [17] | JMIR Serious Games | To elicit perspectives of potential end users on factors related to the acceptability of cognitive training games and assessment technologies. | Four linked workshops were conducted with the same group, each focusing on a specific topic: meaningful improvement, learning and motivation, trust in digital diagnosis, and barriers to technology adoption. | 3 dyads (PwD & carer) | 3 | No |
| Hung, 2023 [18] | Research Involvement and Engagement | To investigate the use of telepresence as an innovative tool for participatory research based on three projects conducted with patient and family partners during the COVID-19 pandemic. | Data sources: (a) team reflective discussions, (b) weekly meeting notes, (c) field notes, and (d) interviews with ten researchers. Patient and family partners contributed to team analysis to identify themes. | 3 PwD and 3 family caregivers | 3 | Yes |
| Kelly, 2025 [19] | Dementia | To present the process of writing the song, the lyrics of the original song, alongside the PPI contributors experiences. | Qualitative research using interpretative phenomenological analysis was used to analyse the process. | PwD supported by caregivers | 3 | Yes |
| Liddle, 2022 [20] | Australian Occupational Therapy Journal | To capture the requirements, actions, and barriers experienced in forming partnerships with PwD in technology research and design. | Meta-reflection of experiences by 5 members of the research teams involved in three participatory research and technology design studies. | 5: 1 person with AD and their spouse, 1 person with FTD, 2 researchers | 2 | Yes |
| Litherland, 2018 [21] | Dementia | The article describes the work of an involvement group, the methods used and their experiences of being involved and the impact it has had, for themselves and others. | The article has been written following a reflective piece of work with the ‘Action on Living Well: Asking You’ group to review and remember their work. | Rolling membership, 4 PwD and five carers or former carers who contribute regularly. Other PwD and carers have been involved in occasional meetings to discuss additional study materials. | 4 | Yes |
| Mann, 2019 [22] | Action Research | The paper presents shared experiences (a person with dementia and a researcher) gained from action research, titled ‘Co-creating Person-Centred Care in Acute Care’. | Action research | Person with Alzheimer's as co-researcher | 1 | Yes |
| Masoud, 2021 [23] | Research Involvement and Engagement | To describe experiences of a stakeholder advisory council members engaged in the research process. | Thematic analysis of semi-structured interviews using an inductive-deductive approach. | 2 PwD, 4 family care partners, 5 health and social care professionals in dementia care | 2 | Yes |
| Miah, 2020a (Training) [24] | Health Expectations | To evaluate the acceptability and perceived outcomes of the training from the perspective of Research User Group members. | Completing the Training Acceptability Rating Scale & semi-structured interviews | 20 PwD, 14 care partners | 20 | Yes |
| Miah, 2020b (Impact) [25] | BMJ Open | To evaluate the impact of PPI at each stage of the research cycle in a dementia research programme. | Monitoring forms & qualitative interview analysis. | 11 PwD, 12 care partners, 9 researchers and 4 project managers | (11 - same group as above) | Yes |
| Middleton, 2025 [26] | Dementia | To describe the participatory approach used to co-design the Dementia Lifestyle Intervention for Getting Healthy Together (DELIGHT) program | Six online, 90 min co-design meetings. Use of breakout rooms for discussion, with PwD in each breakout room. | Co-design team included PwD, care partners, exercise professionals, dietitians, health care professionals, and community service providers (n = 29) | 6 | Yes |
| Micklewright, 2024 [27] | Research Involvement and Engagement | To report on a novel approach to PPIE, developed in response to Covid-19, and utilised in a large UK-based study focused on care homes. | Exploratory design to see if it is possible to support “in-house” PPIE, with researchers working at a distance in partnership with care home staff. | 5 activity providers as PPIE-facilitators 🡪 population of study sites were invited | Unclear | No |
| Poland, 2019 [28] | Health Expectations | To critically evaluate the implementation of PPI activity, including co‐research in a programme of research exploring ways to enhance the independence of PwD. | Visible and explicate theoretical and moral challenges of PPI using critical cases. | 3 PPI with lived experience for the study itself, workshop groups in case 3 | Unclear | Yes |
| Rai 2020 [29] | JMIR Aging | To develop and investigate the usability of Thinkability, an iCST app that can be used by people with dementia and carers on touchscreen tablets. | Agile approach with elements from action research. Developed in 3 successive sprints and evaluation by relevant stakeholders at each sprint. | PwD and caregivers | 2 | No |
| Reid, 2025 [30] | Leisure Sciences | To explore how individuals living with dementia make sense of their lived experiences individually and collectively. | Community-Based Participatory Research with arts-based qualitative methods | 7 people with early-stage dementia | 7 | No |
| Robinson, 2009 [31] | International Psychogeriatrics | To create acceptable and effective prototype technologies to facilitate independence for PwD through a user-centred design process | Three-stage participatory design process: Scoping stage, participatory design stage, prototype development stage. | Scoping stage (5 focus groups, 10 PwD and 11 carers); participatory design stage (5 workshops, 22 participants) and prototype development stage (4 meetings with 2 PwD and 1 carer). | 12 | No |
| Sakamoto, 2023 [32] | International Journal of Geriatric Psychiatry | To discuss and critically reflect on the experiences of patients/residents, family partners, and healthcare staff involved in the co‐design process of an app. | Participatory co-design approach. Interviews analysed using reflexive thematic analysis. | Research team consisted of academic and disciplinary researchers in Nursing, Engineering, Computer Science, and Health Design. This also included patient partners, including family caregivers and a person with lived experience of dementia. Workshops with end-users. | 9 | Yes |
| Sandoval, 2024 [33] | Journal of the American Geriatrics Society | To prioritise emergency care research opportunities for PwD, inclusive of perspectives of PwD, to ensure identification of research gaps in response to their experiences and priorities. | Best practices for supporting the engagement of PwD in a virtual consensus conference (modified Delphi-approach), applied across its three phases (pre-conference, during the conference, and post-conference). | 2 Co-PIs, 2 project managers, 1 dementia advocate specialist, 4 PwD and 2 care partners | 6 | Yes |
| Smith, 2023 [34] | Nurse Researcher | To reflect on establishing an advisory group for a doctoral study, exploring the opportunities and challenges associated with including patients with dementia in the research process. | The authors discuss the practicalities of establishing an advisory group, the challenges of being a novice researcher, long-term commitment to PPI, the overall approach to PPI and ethical considerations. | 3 PwD, 1 family caregiver, 1 healthcare professional working with PwD | 3 | Yes |
| Snowball, 2022 [35] | Research Involvement and Engagement | To describe activities, challenges and mitigation of an advisory group and cross-cutting program. | Descriptive methods | 5 PwD, 12 (former) caregivers | 5 | Yes |
| Stevenson, 2019 [36] | Dementia | To report an exercise involving individuals with dementia as co-researchers in a qualitative analysis. | Co-researchers were involved in deriving meaning from the data, identifying and connecting themes. The analysis process is described, reflections on the exercise provided and impact discussed. | 4 PwD | 4 | Yes |
| Tanner, 2012 [37] | Journal of Mental Health | To discuss the implications for PwD of involvement in research as co-researchers. | Older PwD participated in planning the research methods, conducting interviews and making sense of the findings. | 3 PwD | 3 | Yes |
| Thoft, 2020 [38] | Dementia | To explore how to work collaboratively with PwD to develop their own research projects. | Design of a participatory research model to support PwD in research based on a participatory research project. | 12 people with early onset dementia, divided into two groups (n = 6) | 12 | Yes |
| Waite, 2019 [39] | Health Expectations | To discover the facilitators of, and barriers to, involving PwD as co‐researchers, from different perspectives. | Thematic analysis of data from individual interviews about the co‐research experience. | 6 academic researchers, 8 gatekeepers and 5 PwD | 5 | Yes |
| Warran, 2024 [40] | Arts & Health | To create a collaborative research agenda for dance in dementia. | In-person workshop, online workshop, digital communications, podcast and co-writing. | PwD, family carers health and social care professionals, artists, arts organisational representatives, academic researchers | 19 | Yes |
| Watson, 2023 [41] | Dementia | To develop, implement, and evaluate a face-face programme of support with, by and for people with direct experience of dementia. | Three-phase participatory research project. This paper focuses on the participatory approaches used in development. | 40 PwD, 26 care partners, 31 health and social care practitioners | 40 | Yes |
| Wiersma, 2016 [42] | Dementia | Analysis of discussions of whether or not PwD should work in segregated groups within a research project. | Thematic analysis of ongoing discussions. | 20 PwD, 13 care partners and 3 service providers | 20 | Yes |

**Abbreviations**: AD: Alzheimer’s dementia, app: mobile application, COVID-19: Coronavirus disease 2019, FTD: frontotemporal dementia, GRIPP2: Guidance for Reporting Involvement of Patients and the Public version 2, iCST: individual cognitive stimulation therapy, JMIR: Journal of Medical Internet Research, n: sample size, NGO: non-governmental organisation, PAR: participatory action research, PI: principal investigator, PPI: patient and public involvement, PPIE: patient and public involvement and engagement, PwD: person/people with dementia, QoL: quality of life, UK: United Kingdom

**Weight-of-evidence ratings^1^**

| **Study (Author, year)** | **Design** | **Quality of execution of study** | **Appropriateness of method** | **Focus/approach of study to review question** | **Overall rating** | **Comment** |
| --- | --- | --- | --- | --- | --- | --- |
| Beresford-Dent, 2022 [1] | Reflexive qualitative analysis (using GRIPP2-Short) | high | high | high | high | Reporting is transparent and aligned with GRIPP2 guidelines, demonstrating high methodological rigour and strong thematic relevance for PPIE in dementia research. |
| Bielsten, 2018 [2] | Collaborative research | high | low | medium | low | The study is of good overall quality; however, its primary focus lies at the service level. PPI activities are neither systematically reflected upon nor formally evaluated. Some methodological details are provided regarding the recruitment of people with dementia and the accommodation of their needs. |
| Bloska, 2024 [3] | Survey study with qualitative analysis, GRIPP2 checklist | high | medium | medium | medium | The study is methodologically robust; however, it primarily addresses general experiences of international PPIE collaboration rather than detailing specific recruitment strategies or modes of involvement for PwD. |
| Bosco, 2019 [4] | Meta-ethnographic study | high | low | medium | low | Despite its high methodological quality, the study engages insufficiently with PPI aspects. Co-production is conceptualised within a care context rather than as active involvement of PwD in the research process, with the PPI group limited to a consultative role. |
| Brooks, 2017 [5] | Qualitative descriptive and mixed-methods study | high | medium | medium | medium | The study offers practical insights into recruitment, consent, and organisational challenges; however, it addresses PPIE only selectively and lacks continuous involvement of PwD throughout the research process, resulting in moderate relevance to the research questions. |
| Capstick, 2021 [6] | Qualitative co-creation study | high | high | high | high | The study provides a detailed account of innovative and adaptive recruitment and involvement strategies, alongside sustained co-creation with PwD throughout all stages of the research process. |
| Clarke, 2018 [7] | Participatory secondary qualitative data analysis | high | high | high | high | The study adopts a participatory research approach, actively involving PwD as co-analysts throughout the data analysis process. It thus offers directly relevant insights into forms of involvement of this population in research. |
| Clarke, 2020 [8] | Participatory secondary qualitative data analysis (results paper) | high | low | medium | low | The study primarily focuses on the further development of a care model and addresses PPI processes only marginally. However, due to its relation to Clarke (2018) [7], it was included in this systematic review but not in the meta-synthesis. |
| de Wolf-Linder, 2024 [10] | Qualitative thematic data analysis | high | medium | low | low | The study offers information on the dissemination phase; however, only a small number of PwD are involved, and their participation in the research process itself is not examined. |
| Di Lorito, 2024 [10] | Descriptive article on co-design activities with PwD | high | high | medium | high | The study presents a clearly structured and well-documented PPIE process involving underserved communities, detailing both recruitment strategies and specific forms of involvement across multiple project phases. Its overall fit was rated as moderate due to its primary focus on website development.. |
| Ditton, 2025 [11] | Descriptive article and reflection on PPIE activities in a doctoral project | high | high | high | high | The study provides a detailed account of a transparent and reflexive PPIE process involving one PwD and their supporter, including the recruitment pathway, the organisation of collaboration, and specific contributions to research planning. |
| Donnelly, 2024 [12] | End-user testing and feedback from focus groups | medium | medium | medium | medium | The study outlines a structured PPIE process for developing a person-centred survey on dementia with Lewy bodies, in which affected individuals and family carers were selectively involved in item development. However, the primary emphasis is on questionnaire optimisation, while recruitment strategies and broader forms of involvement across the full research process are only described to a limited extent. In addition, analytical methods are not clearly reported. |
| Dooley, 2022 [13] | Reflexive analysis of the involvement of people with dementia in data analysis | high | high | high | high | The study demonstrates a clearly structured process in which PwD are actively involved in the analysis of video and transcript data, making substantial contributions to the research process. Recruitment pathways and adaptations to the analytical format are reported transparently; however, the primary focus remains on participatory data analysis. |
| Dupuis, 2021 [14] | Participatory Action Research | high | medium | high | medium | The study outlines a participatory development process but does not provide detailed accounts of how PwD were concretely involved at each stage. As a result, involvement is described in general terms, limiting both methodological rigour and thematic depth in relation to the research questions. |
| Fox, 2022 [15] | Iterative co-design process | high | medium | medium | medium | The study describes an iterative co-design process involving workshops and usability testing, in which PwD and family carers contributed feedback on prototypes. However, the involvement of PwD remains relatively superficial, being largely confined to feedback and testing activities. |
| Griffiths, 2024 [16] | Reflexive analysis of diversity in a PPIE group using jointly developed questions | high | medium | medium | medium | The study reflects a co-production approach in the development of a research proposal, involving PwD alongside other stakeholders. However, the specific contributions of PwD are described only in general terms, and details regarding recruitment and participation in the subsequent research process are not provided. |
| Harrington, 2022 [17] | Qualitative thematic analysis of workshop notes | high | low | low | low | The study employs PPI primarily to assess the acceptability of cognitive training games and diagnostic technologies, without detailing concrete forms of involvement of PwD in the research process. Participant roles remain largely consultative, although some information on recruitment and planning is included. |
| Hung, 2023 [18] | Qualitative analysis | high | medium | medium | medium | The study describes the use of a telepresence robot to facilitate participation during the COVID-19 pandemic; however, it focuses predominantly on the technical and organisational aspects of remote involvement rather than on PPI processes. |
| Kelly, 2025 [19] | Qualitative interpretative phenomenological analysis | high | high | high | high | The study implements PPI across the entire research process, involving PwD in priority setting, study development, questionnaire review, recruitment support, and the co-production of dissemination outputs (e.g., songwriting). Contributors are acknowledged as co-authors, and their perspectives are systematically analysed. |
| Liddle, 2022 [20] | Meta-reflection of a PPI group | high | high | high | high | The study describes a continuous and well-documented PPIE process in which PwD and family carers are actively involved across multiple phases, including planning, prioritisation, and the design of technological solutions. Recruitment pathways, barriers, and adaptations are reported transparently, and contributions from PwD demonstrably influenced methodological and design decisions. |
| Litherland, 2018 [21] | Reflection on a PPI group | medium | high | high | high | The study describes a long-established PPIE framework featuring continuous and diverse involvement of PwD and family carers in the development of materials and instruments, as well as in analysis and dissemination. Recruitment processes, support structures, and participant roles are reported transparently, with the contributions of the study group having a clear influence on study design and conduct. However, the study is presented primarily as an experience-based report. |
| Mann, 2019 [22] | Reflection by one person with dementia and one researcher on a shared research process | medium | high | high | high | The study presents a comprehensive, methodologically grounded action research project in which PwD are actively engaged as co-researchers across all phases of the research process, including study planning, data collection, reflective processes, knowledge production, and dissemination. Roles, methods, and specific contributions are described and critically reflected in detail. However, the approach is primarily narrative rather than systematically qualitative-analytical. |
| Masoud, 2021 [23] | Qualitative thematic data analysis of semistructured interviews | high | high | medium | medium | The study describes the work of a Stakeholder Advisory Council that included 2 PwD. Engagement strategies and experiences are reported in detail; however, involvement is primarily advisory in nature rather than constituting co-design. |
| Miah, 2020a (Training) [24] | Mixed-methods study (semistructured interviews and analysis of a training measure) | high | high | high | high | The study outlines a structured training programme designed to prepare PwD and family carers for research involvement, while actively incorporating their perspectives into programme development. PPIE components are reported transparently, including recruitment, needs assessment, co-development, and evaluation of the training content. |
| Miah, 2020b (Impact) [25] | Analysis of monitoring forms across the full research process | high | medium | medium | medium | The study reports on the impact of involving PwD and their care partners; however, it does not provide detailed accounts of specific PPIE methods or recruitment procedures. Involvement is discussed primarily at a higher-level perspective and is not systematically analysed across individual stages of the research process. |
| Middleton, 2025 [26] | Descriptive article on co-design workshops for developing an intervention | medium | high | medium | medium | The study describes a series of co-design workshops for the development of a lifestyle programme, in which PwD and family carers were selectively involved. However, detailed accounts of the involvement of PwD across all phases are lacking, and the overall design is primarily narrative. |
| Micklewright, 2024 [27] | Exploratory study design | medium | low | low | low | The study presents an innovative PPIE model in which activity providers act on behalf of care home residents, including individuals with dementia. However, the involvement of PwD is indirect and largely limited to feedback-oriented activities, with no evidence of active participation across multiple research phases. Recruitment and involvement procedures are not systematically analysed. |
| Poland, 2019 [28] | Reflection on PPIE based on three case studies | high | high | medium | medium | The study examines expectations and tensions in PPI processes at a conceptual level, offering valuable theoretical reflections. However, it does not describe a specific PPI process and does not address recruitment procedures. |
| Rai 2020 [29] | Development of an app in three sprints | high | medium | medium | medium | The study outlines an iterative development process in which PwD and family carers primarily provided feedback on app prototypes through PPI consultations and usability testing. Their involvement remains largely consultative, with limited detail on active participation in research decision-making or on recruitment and involvement procedures. |
| Reid, 2025 [30] | Community-based participatory research | medium | low | medium | low | The study provides information on recruitment and co-creation, incorporating arts-based approaches. However, its primary focus lies on how the research object can support PwD rather than on PPI. Consequently, it was not included in the meta-synthesis. |
| Robinson, 2009 [31] | Three-stage participatory design process | medium | low | low | low | The study outlines an early user-centred participatory design approach involving PwD in workshops and focus groups; however, it does not provide a systematic reflection on recruitment or involvement procedures. |
| Sakamoto, 2023 [32] | Co-design of an app using the User Experience Model | high | medium | medium | medium | The study offers a methodologically sound and reflexive account of the co-design process, explicitly addressing PPI aspects. However, the participation and influence of PwD were partially constrained by the pandemic context. While PPI roles across project phases are generally clearly presented, the level of detail is not consistent throughout. |
| Sandoval, 2024 [33] | Qualitative data analysis of semistructured interviews | high | high | high | high | The study provides a detailed and reflexive account of recruitment, preparation, and multi-phase involvement of PwD within a complex consensus conference. PPI processes are described systematically, including adaptations, support strategies, barriers, and facilitators. |
| Smith, 2023 [34] | Narrative reflection | medium | medium | medium | medium | The study offers a reflective account of establishing a PPI advisory group within a doctoral project, including descriptions of recruitment and communication strategies. However, the involvement of PwD remains predominantly advisory and is not systematically documented across multiple research phases. The overall design is narrative-reflective. |
| Snowball, 2022 [35] | Descriptive narrative reporting of PPIE activities from the perspective of researchers and the group | medium | high | high | high | Despite its narrative reporting format, the article provides a clear and transparent account of activities, methodology, challenges, and reflections. The involvement of PwD is substantive, structured, and well documented. |
| Stevenson, 2019 [36] | Experience report and reflection on a PPIE process with people with dementia in qualitative data analysis | medium | high | high | high | The study provides a detailed account of the involvement of PwD as co-researchers in qualitative data analysis, clearly demonstrating how their perspectives informed both interpretation and subsequent data collection. Recruitment processes, methodological approaches, and reflections on the PPI process are reported transparently. |
| Tanner, 2012 [37] | Experience report and reflection on a PPIE process with people with dementia | medium | high | high | high | The study provides a detailed account of the involvement of PwD as co-researchers across multiple phases, including the development of the interview guide, joint conduct of interviews, and data analysis. Recruitment, preparation, support structures, and reflective processes are reported transparently. |
| Thoft, 2020 [38] | Development of a qualitative participatory research model with people with dementia | high | high | high | high | The study describes a comprehensive, methodologically transparent, and multi-phase co-research process involving people with early-stage dementia, encompassing recruitment, training, joint development of research questions, data collection, and data analysis. Involvement is active, substantive, and transparently documented across the full research cycle. |
| Waite, 2019 [39] | Qualitative thematic analysis of interviews on involvement | high | high | high | high | The study presents a systematic and methodologically transparent analysis of facilitators and barriers to co-research with PwD, drawing on interviews with individuals affected, gatekeepers, and researchers. Recruitment and involvement processes are described in detail, alongside structural, ethical, and practical challenges associated with participation. |
| Warran, 2024 [40] | Mixed-methods study | high | high | high | high | The study employs a broad range of methods, including workshops, online workshops, podcasts, follow-up activities, and co-writing, with PwD actively involved across multiple phases. Participant roles are clearly defined, the process is transparently reported, and PPI constitutes a central element of the overall research framework. |
| Watson, 2023 [41] | Participatory Action Research | high | high | high | high | The PPI process is described in detail, encompassing co-analysis, shared decision-making, and continuous reflection on barriers and facilitating factors. |
| Wiersma, 2016 [42] | Descriptive narrative report with reflection and analysis of PPI activities | high | high | high | high | The study offers an empirically grounded and theoretically robust analysis of how group structures shape the participation and opportunities for expression of PwD. Active involvement is described in detail, alongside facilitating and constraining conditions, facilitation strategies, and structural factors influencing participation. |

**Abbreviations**: COVID-19: Coronavirus disease 2019, GRIPP2: Guidance for Reporting Involvement of Patients and the Public version 2, PPI: patient and public involvement, PPIE: patient and public involvement and engagement, PwD: person/people with dementia, WoE: weight of evidence

**^1^Weight-of-evidence ratings**: Gough’s weight of evidence rating [42] assesses the following aspects:

(1) Quality of execution of study: assesses the internal methodological quality of the study in its own terms (i.e., how well it was conducted, independent of the review question). Ratings are assigned as: (i) high: methodologically rigorous, transparent, and coherent; low risk of bias, (ii) medium: adequate quality with some limitations in design, execution, or reporting, (iii) low: significant methodological weaknesses or poor reporting, limiting trustworthiness.

(2) Appropriateness of method: assesses the fit of the study design and methods for answering the review question (fitness for purpose). Ratings are assigned as: (i) high: study design is highly appropriate for addressing the specific review question, (ii) medium: design is partially appropriate but with some limitations for the question, (iii) low: design is poorly suited to address the review question.

(3) Focus/approach of study to review question: assesses the relevance of the study’s topic, sample, and context to the review question. Ratings are assigned as: (i) high: directly addresses the review question (relevant population, context, outcomes), (ii) medium: indirect or partial relevance to the review question, (iii) low: limited relevance (e.g., different population, context, or focus).

(4) Overall rating: represents the combined judgement of weight of evidence A–C, indicating the extent to which the study contributes to answering the review question. Ratings are assigned as: (i) high: strong contribution; both high quality and high relevance, (ii) medium: moderate contribution; some limitations in quality and/or relevance, (iii) low: limited contribution due to methodological weaknesses and/or low relevance

**References**

[1] J. Beresford-Dent, K. Sprange, G. Mountain, et al., “Embedding patient and public involvement in dementia research: Reflections from experiences during the ‘Journeying through Dementia’ randomised controlled trial,” *Dementia* 21, no. 6 (2022): 1987-2003, <https://doi.org/10.1177/14713012221106816>.

[2] T. Bielsten, R. Lasrado, J. Keady, A. Kullberg, and I. Hellström, “Living life and doing things together: collaborative research with couples where one partner has a diagnosis of dementia,” *Qualitative Health Research* 28, no. 11 (2018):1719-1734, <https://doi.org/10.1177/1049732318786944>.

[3] J. Bloska, S. Crabtree, N. Wollersberger, et al., “Experiences of participant and public involvement in an international randomized controlled trial for people living with dementia and their informal caregivers,” *Research Involvement & Engagement* 10, no. 1 (2024): 43, <https://doi.org/10.1186/s40900-024-00574-2>.

[4] A. Bosco, J. Schneider, D. M. Coleston-Shields, and M. Orrell, “Dementia care model: promoting personhood through co-production,” *Archives of Gerontology & Geriatrics* 81 (2019): 59-73, <https://doi.org/10.1016/j.archger.2018.11.003>.

[5] J. Brooks, K. Gridley, and N. Savitch, “Removing the ‘gag’: involving people with dementia in research as advisers and participants,” *Social Research Practice* 201, no. 3 (2017): 3-14.

[6] A. Capstick, A. Dennison, J. Oyebode, et al., “Drawn from life: Cocreating narrative and graphic vignettes of lived experience with people affected by dementia,” Health Expectations 24, no. 5 (2021): 1890-1900, <https://doi.org/10.1111/hex.13332>.

[7] C. L. Clarke, H. Wilkinson, J. Watson, J. Wilcockson, L. Kinnaird, and T. Williamson, “A seat around the table: participatory data analysis with people living with dementia,” *Qualitative Health Research* 28, no. 9 (2018): 1421-1433, <https://doi.org/10.1177/1049732318774768>.

[8] C. L. Clarke, J. Wilcockson, J. Watson, et al., “Relational care and co-operative endeavour - Reshaping dementia care through participatory secondary data analysis,” *Dementia (London)* 19, no. 4 (2020): 1151-1172, <https://doi.org/10.1177/1471301218795353>.

[9] S. de Wolf-Linder, I. Kramer, M. Hersperger, et al., “Meaningful patient and public engagement in dissemination-embedding co-production in dementia research,” *Frontiers in Dementia* 3 (2024): 1426019, <https://doi.org/10.3389/frdem.2024.1426019>.

[10] C. Di Lorito, S. Griffiths, M. Poole, et al., “Patient and public involvement and engagement with underserved communities in dementia research: Reporting on a partnership to co-design a website for postdiagnostic dementia support,” *Health Expectations* 21, no. 1 (2024): e13992, <https://doi.org/10.1111/hex.13992>.

[11] A. Ditton, D. Bunt, and P. Berry, “A multi-person reflection on using public and patient involvement and engagement to develop dementia research,” *Research Involvement and Engagement* 11, no. 1 (2025): 8, <https://doi.org/10.1186/s40900-025-00678-3>.

[12] P. S. Donnelly, A. Sweeney, E. Wilson, et al., “Developing a person-centered stated preference survey for dementia with Lewy bodies: value of a personal and public involvement process,” *Frontiers in Dementia* 3 (2024): 1421556, <https://doi.org/10.3389/frdem.2024.1421556>.

[13] J. Dooley, “Involving people with experience of dementia in analysis of video recorded doctor-patient-carer interactions in care homes,” *International Journal of Social Research Methodology* 25, no. 1 (2022): 1-13, <https://doi.org/10.1080/13645579.2020.1826648>.

[14] S. Dupuis, C. McAiney, L. Loiselle, B. Hounam, J. Mann, and E. C. Wiersma, “Use of participatory action research approach to develop a self-management resource for persons living with dementia,” *Dementia (London)* 20, no. 7 (2021): 2393-2411, <https://doi.org/10.1177/1471301221997281>.

[15] S. Fox, L. J. E. Brown, S. Antrobus, et al., “Co-design of a smartphone app for people living with dementia by applying agile, iterative co-design principles: development and usability study,” *JMIR mHealth and uHealth* 10, no. 1 (2022): e24483, <https://doi.org/10.2196/24483>.

[16] S. Griffiths, M. Robertson, C. Kaviraj, et al., “Reflections on co-production: Developing a dementia research funding application with a diverse lived experience group,” *Dementia (London)* 23, no. 5 (2024): 709-723, <https://doi.org/10.1177/14713012241231916>.

[17] K. Harrington, M. P. Craven, M. L. Wilson, and A. Landowska, “Perceptions of cognitive training games and assessment technologies for dementia: acceptability study with patient and public involvement workshops,” *JMIR Serious Games* 10, no. 2 (2022): e32489, <https://doi.org/10.2196/32489>.

[18] L. Hung, C. Lake, A. Hussein, J. Wong, and J. Mann, “Using telepresence robots as a tool to engage patient and family partners in dementia research during COVID-19 pandemic: a qualitative participatory study,” *Research Involvement and Engagement* 9, no. 1 (2023): 12, <https://doi.org/10.1186/s40900-023-00421-w>.

[19] L. Kelly, C. Corcoran, G. Paley, et al., “’We will tell everyone!’ Capturing the impact of public and patient involvement in music therapy and dementia research through songwriting,” *Dementia (London)* 24, no. 8 (2025): 1600-1614, <https://doi.org/10.1177/14713012251333839>.

[20] J. Liddle, P. Worthy, D. Frost, E. Taylor, and D. Taylor, “Partnering with people living with dementia and care partners in technology research and design: Reflections and recommendations,” *Australian Occupational Therapy Journal* 69, no. 6 (2022): 723-741, <https://doi.org/10.1111/1440-1630.12843>.

[21] R. Litherland, J. Burton, M. Cheeseman, et al., “Reflections on PPI from the ‘Action on Living Well: Asking You’ advisory network of people with dementia and carers as part of the IDEAL study,” *Dementia (London)* 17, no 8. (2018): 1035-1044, <https://doi.org/10.1177/1471301218789309>.

[22] J. Mann, and L. Hung, “Co-research with people living with dementia for change,” *Action Research* 17, no. 4 (2019): 573-590, <https://doi.org/10.1177/1476750318787005>.

[23] S. S. Masoud, A. A. Glassner, N. Patel, et al., “Engagement with a diverse Stakeholder Advisory Council for research in dementia care,” *Research Involvement and Engagement* 7, no. 1 (2021): 54, <https://doi.org/10.1186/s40900-021-00297-8>.

[24] J. Miah, S. Parsons, K. Lovell, B. Starling, I. Leroi, and P. Dawes, “Impact of involving people with dementia and their care partners in research: a qualitative study,” *BMJ Open* 10, no. 10 (2020): e039321, <https://doi.org/10.1136/bmjopen-2020-039321>.

[25] J. Miah, P. Dawes, I. Leroi, et al., “Evaluation of a research awareness training programme to support research involvement of older people with dementia and their care partners,” *Health Expectations* 23, no. 5 (2020): 1177-1190, <https://doi.org/10.1111/hex.13096>.

[26] L. E. Middleton, L. Bechard, W. Heibein, et al., “The co-design of the Dementia Lifestyle Intervention to get healthy together (DELIGHT) program: An authentic partnership approach,” *Dementia (London)* 25, no. 13 (2025): 14713012251325928, <https://doi.org/10.1177/14713012251325928>.

[27] K. Micklewright, A. Killett, G. Akdur, et al., “Activity provider-facilitated patient and public involvement with care home residents,” *Research Involvement and Engagement* 10, no. 1 (2024): 7, <https://doi.org/10.1186/s40900-023-00537-z>.

[28] F. Poland, G. Charlesworth, P. Leung, and L. Birt. “Embedding patient and public involvement: Managing tacit and explicit expectations,” *Health Expectations* 22, no. 6 (2019): 1231-1239, <https://doi.org/10.1111/hex.12952>.

[29] H. K. Rai, J. Schneider, and M. Orrell, “An individual cognitive stimulation therapy app for people with dementia: development and usability study of thinkability,” *JMIR Aging* 3, no. 2 (2020): e17105, <https://doi.org/10.2196/17105>.

[30] C. Reid, A. Landy, and J. Henderson, “From ‘the tragic self’ to ‘the empowered self’: how leisure spaces can foster narrative agency for people with lived experience of dementia,” *Leisure Sciences* (2025): 1–22, <https://doi.org/10.1080/01490400.2025.2454524>.

[31] L. Robinson, K. Brittain, S. Lindsay, D. Jackson, and P. Olivier, “Keeping In Touch Everyday (KITE) project: developing assistive technologies with people with dementia and their carers to promote independence,” *International Journal of Psychogeriatrics* 21, no. 3 (2009): 494-502, <https://doi.org/10.1017/S1041610209008448>.

[32] M. Sakamoto, Y. P. Guo, K. L. Y. Wong, et al., “Co‐design of a digital app “WhatMatters” to support person‐centred care: A critical reflection,” *International Journal of Geriatric Psychiatry* 38, no. 10 (2023): e6014, <https://doi.org/10.1002/gps.6014>.

[33] J. Sandoval, A. Gilmore-Bykovskyi, C. R. Carpenter, et al., “Moving beyond tokenism: Sustaining engagement of persons living with dementia in identifying emergency research priorities,” *Journal of the American Geriatrics Society* 73, no. 5 (2025): 1344-1352, <https://doi.org/10.1111/jgs.19269>.

[34] C. Smith, J. Baillie, and P. Gill, “Importance of patient and public involvement in doctoral research involving people living with dementia,” *Nurse Researcher* 32, no. 2 (2024): 39-45, <https://doi.org/10.7748/nr.2024.e1919>.

[35] E. Snowball, R. Fernandez Loughlin, H. Eagleson, et al., “Engagement of people with lived experience of dementia advisory group and cross-cutting program: reflections on the first year,” Research Involvement and Engagement 8, no. 1 (2022): 28, <https://doi.org/10.1186/s40900-022-00359-5>.

[36] M. Stevenson, and B. J. Taylor, “Involving individuals with dementia as co-researchers in analysis of findings from a qualitative study,” *Dementia (London)* 18, no. 2 (2019): 701-712, <https://doi.org/10.1177/1471301217690904>.

[37] D. Tanner, “Co-research with older people with dementia: experience and reflections,” *Journal of Mental Health* 21, no. 3 (2012): 296-306, <https://doi.org/10.3109/09638237.2011.651658>.

[38] D. S. Thoft, M. Pyer, A. Horsbøl, and J. Parkes, “The Balanced Participation Model: Sharing opportunities for giving people with early-stage dementia a voice in research,” *Dementia (London)* 19, no. 7 (2020): 2294-2313, <https://doi.org/10.1177/1471301218820208>.

[39] J. Waite, F. Poland, and G. Charlesworth, “Facilitators and barriers to co‐research by people with dementia and academic researchers: Findings from a qualitative study,” *Health Expectations* 22, no. 4 (2019): 761-771, <https://doi.org/10.1111/hex.12891>.

[40] K. Warran, P. Letrondo, M. Robertson, et al., “What are the research priorities in dance for dementia? A co-created agenda to support equitable research,” *Arts and Health* (2024): 1-23, <https://doi.org/10.1080/17533015.2024.2408023>.

[41] J. Watson, J. Wilcockson, A. Houston, et al., “'I feel more part of the world': Participatory action research to develop post-diagnostic dementia support,” *Dementia (London)* 22, no. 7 (2023): 1420-1439, <https://doi.org/10.1177/14713012231190775>.

[42] E. C. Wiersma, D. L. O’Connor, L. Loiselle, et al., “Creating space for citizenship: The impact of group structure on validating the voices of people with dementia,” *Dementia (London)* 15, no. 3 (2016): 414-433, <https://doi.org/10.1177/1471301216642339>.
