# Supplementary material for: Involving People With Dementia in Research: Contributions, Barriers and Evaluation From a Meta‐Synthesis and Qualitative Interview Study
Source: Health Expect. 2026 Sep 7;29(5):e70864. doi: 10.1111/hex.70864 (PMC13548409; doi:10.1111/hex.70864)
Supplement: Supplementary file 2 [file HEX-29-e70864-s002.docx]

**Meta-synthesis – methods for meaningful involvement of PwD in different research studies**

| **Research phase** | **Specific methods for involving PwD** |
| --- | --- |
| Pre-trial | - PPIE/advisory groups [1-3] - Co-design of study / intervention and study materials [1, 3-6] - Collaborative priority-setting or development of research questions [3, 7-9] - Training and clarification of roles as co-researchers [5, 10-13] - Involvement in ethical and organisational study planning [3-5] |
| Recruitment | - Advice on recruitment and support materials [4] |
| Data collection | - Focus groups and discussion (including online and semi-structured formats) [7, 10, 14-17] - (Co-)Interviews face-to-face or by telephone using cue cards and jointly developed interview guides [3, 13, 18] - Participatory workshops with PwD as experts and/or facilitators [3, 5] - (Online-)surveys/questionnaires, developed and piloted iteratively with PwD [1] - Joint reflection and feedback rounds, with ongoing adaptation of the setting, materials and communication [1, 3, 4, 13, 19-21] |
| Data analysis | - Collaborative interpretation of interview data in workshops/advisory groups using dementia-friendly materials [3, 20, 22-25] - Narrative and creative methods (stories, images, cards) for interpreting data [12, 16, 20, 23, 24] - Iterative feedback and revision rounds on categories/themes [26-28] |
| Publication & dissemination | - Co-authorship by people with dementia [3, 20, 26] - Joint development of easy-to-understand materials (flyers, reports, infographics) [1, 3, 9, 22, 26, 29] - Co-presentation at events [5, 18] - Involvement in videos, blogs, social media and local information events [1, 11, 22, 30] |

**Abbreviations**: PwD: person with dementia

**References**

[1] J. Bloska, S. Crabtree, N. Wollersberger, et al., “Experiences of participant and public involvement in an international randomized controlled trial for people living with dementia and their informal caregivers,” *Research Involvement & Engagement* 10, no. 1 (2024): 43, <https://doi.org/10.1186/s40900-024-00574-2>.

[2] S. Griffiths, M. Robertson, C. Kaviraj, et al., “Reflections on co-production: Developing a dementia research funding application with a diverse lived experience group,” *Dementia (London)* 23, no. 5 (2024): 709-723, <https://doi.org/10.1177/14713012241231916>.

[3] D. S. Thoft, M. Pyer, A. Horsbøl, and J. Parkes, “The Balanced Participation Model: Sharing opportunities for giving people with early-stage dementia a voice in research,” *Dementia (London)* 19, no. 7 (2020): 2294-2313, <https://doi.org/10.1177/1471301218820208>.

[4] A. Ditton, D. Bunt, and P. Berry, “A multi-person reflection on using public and patient involvement and engagement to develop dementia research,” *Research Involvement and Engagement* 11, no. 1 (2025): 8, <https://doi.org/10.1186/s40900-025-00678-3>.

[5] J. Mann, and L. Hung, “Co-research with people living with dementia for change,” *Action Research* 17, no. 4 (2019): 573-590, <https://doi.org/10.1177/1476750318787005>.

[6] C. Smith, J. Baillie, and P. Gill, “Importance of patient and public involvement in doctoral research involving people living with dementia,” *Nurse Researcher* 32, no. 2 (2024): 39-45, <https://doi.org/10.7748/nr.2024.e1919>.

[7] J. Brooks, K. Gridley, and N. Savitch, “Removing the ‘gag’: involving people with dementia in research as advisers and participants,” *Social Research Practice* 201, no. 3 (2017): 3-14.

[8] J. Sandoval, A. Gilmore-Bykovskyi, C. R. Carpenter, et al., “Moving beyond tokenism: Sustaining engagement of persons living with dementia in identifying emergency research priorities,” *Journal of the American Geriatrics Society* 73, no. 5 (2025): 1344-1352, <https://doi.org/10.1111/jgs.19269>.

[9] E. Snowball, R. Fernandez Loughlin, H. Eagleson, et al., “Engagement of people with lived experience of dementia advisory group and cross-cutting program: reflections on the first year,” Research Involvement and Engagement 8, no. 1 (2022): 28, <https://doi.org/10.1186/s40900-022-00359-5>.

[10] C. Di Lorito, S. Griffiths, M. Poole, et al., “Patient and public involvement and engagement with underserved communities in dementia research: Reporting on a partnership to co-design a website for postdiagnostic dementia support,” *Health Expectations* 21, no. 1 (2024): e13992, <https://doi.org/10.1111/hex.13992>.

[11] R. Litherland, J. Burton, M. Cheeseman, et al., “Reflections on PPI from the ‘Action on Living Well: Asking You’ advisory network of people with dementia and carers as part of the IDEAL study,” *Dementia (London)* 17, no 8. (2018): 1035-1044, <https://doi.org/10.1177/1471301218789309>.

[12] J. Miah, P. Dawes, I. Leroi, et al., “Evaluation of a research awareness training programme to support research involvement of older people with dementia and their care partners,” *Health Expectations* 23, no. 5 (2020): 1177-1190, <https://doi.org/10.1111/hex.13096>.

[13] D. Tanner, “Co-research with older people with dementia: experience and reflections,” *Journal of Mental Health* 21, no. 3 (2012): 296-306, <https://doi.org/10.3109/09638237.2011.651658>.

[14] P. S. Donnelly, A. Sweeney, E. Wilson, et al., “Developing a person-centered stated preference survey for dementia with Lewy bodies: value of a personal and public involvement process,” *Frontiers in Dementia* 3 (2024): 1421556, <https://doi.org/10.3389/frdem.2024.1421556>.

[15] S. Fox, L. J. E. Brown, S. Antrobus, et al., “Co-design of a smartphone app for people living with dementia by applying agile, iterative co-design principles: development and usability study,” *JMIR mHealth and uHealth* 10, no. 1 (2022): e24483, <https://doi.org/10.2196/24483>.

[16] L. Kelly, C. Corcoran, G. Paley, et al., “’We will tell everyone!’ Capturing the impact of public and patient involvement in music therapy and dementia research through songwriting,” *Dementia (London)* 24, no. 8 (2025): 1600-1614, <https://doi.org/10.1177/14713012251333839>.

[17] J. Watson, J. Wilcockson, A. Houston, et al., “'I feel more part of the world': Participatory action research to develop post-diagnostic dementia support,” *Dementia (London)* 22, no. 7 (2023): 1420-1439, <https://doi.org/10.1177/14713012231190775>.

[18] J. Miah, S. Parsons, K. Lovell, B. Starling, I. Leroi, and P. Dawes, “Impact of involving people with dementia and their care partners in research: a qualitative study,” *BMJ Open* 10, no. 10 (2020): e039321, <https://doi.org/10.1136/bmjopen-2020-039321>.

[19] J. Liddle, P. Worthy, D. Frost, E. Taylor, and D. Taylor, “Partnering with people living with dementia and care partners in technology research and design: Reflections and recommendations,” *Australian Occupational Therapy Journal* 69, no. 6 (2022): 723-741, <https://doi.org/10.1111/1440-1630.12843>.

[20] M. Stevenson, and B. J. Taylor, “Involving individuals with dementia as co-researchers in analysis of findings from a qualitative study,” *Dementia (London)* 18, no. 2 (2019): 701-712, <https://doi.org/10.1177/1471301217690904>.

[21] L. Hung, C. Lake, A. Hussein, J. Wong, and J. Mann, “Using telepresence robots as a tool to engage patient and family partners in dementia research during COVID-19 pandemic: a qualitative participatory study,” *Research Involvement and Engagement* 9, no. 1 (2023): 12, <https://doi.org/10.1186/s40900-023-00421-w>.

[22] J. Beresford-Dent, K. Sprange, G. Mountain, et al., “Embedding patient and public involvement in dementia research: Reflections from experiences during the ‘Journeying through Dementia’ randomised controlled trial,” Dementia 21, no. 6 (2022): 1987-2003, <https://doi.org/10.1177/14713012221106816>.

[23] C. L. Clarke, H. Wilkinson, J. Watson, J. Wilcockson, L. Kinnaird, and T. Williamson, “A seat around the table: participatory data analysis with people living with dementia,” *Qualitative Health Research* 28, no. 9 (2018): 1421-1433, <https://doi.org/10.1177/1049732318774768>.

[24] J. Dooley, “Involving people with experience of dementia in analysis of video recorded doctor-patient-carer interactions in care homes,” *International Journal of Social Research Methodology* 25, no. 1 (2022): 1-13, <https://doi.org/10.1080/13645579.2020.1826648>.

[25] F. Poland, G. Charlesworth, P. Leung, and L. Birt. “Embedding patient and public involvement: Managing tacit and explicit expectations,” *Health Expectations* 22, no. 6 (2019): 1231-1239, <https://doi.org/10.1111/hex.12952>.

[26] A. Capstick, A. Dennison, J. Oyebode, et al., “Drawn from life: Cocreating narrative and graphic vignettes of lived experience with people affected by dementia,” Health Expectations 24, no. 5 (2021): 1890-1900, <https://doi.org/10.1111/hex.13332>.

[27] S. S. Masoud, A. A. Glassner, N. Patel, et al., “Engagement with a diverse Stakeholder Advisory Council for research in dementia care,” *Research Involvement and Engagement* 7, no. 1 (2021): 54, <https://doi.org/10.1186/s40900-021-00297-8>.

[28] E. C. Wiersma, D. L. O’Connor, L. Loiselle, et al., “Creating space for citizenship: The impact of group structure on validating the voices of people with dementia,” *Dementia (London)* 15, no. 3 (2016): 414-433, <https://doi.org/10.1177/1471301216642339>.

[29] S. Dupuis, C. McAiney, L. Loiselle, B. Hounam, J. Mann, and E. C. Wiersma, “Use of participatory action research approach to develop a self-management resource for persons living with dementia,” *Dementia (London)* 20, no. 7 (2021): 2393-2411, <https://doi.org/10.1177/1471301221997281>.

[30] K. Warran, P. Letrondo, M. Robertson, et al., “What are the research priorities in dance for dementia? A co-created agenda to support equitable research,” *Arts and Health* (2024): 1-23, <https://doi.org/10.1080/17533015.2024.2408023>.
