# Supplementary material for: Involving People With Dementia in Research: Contributions, Barriers and Evaluation From a Meta‐Synthesis and Qualitative Interview Study
Source: Health Expect. 2026 Sep 7;29(5):e70864. doi: 10.1111/hex.70864 (PMC13548409; doi:10.1111/hex.70864)
Supplement: Supplementary file 3 [file HEX-29-e70864-s003.docx]

**Operationalisation of research question 1 (part 1, meta-synthesis)**

**Aim (1)**: To identify and analyse procedures and strategies that facilitate meaningful involvement of PwD in PPIE across different phases of the research cycle.

| Research question 1a: Which procedures and techniques contribute to the recruitment and involvement of people with dementia in PPIE groups? | |
| --- | --- |
| Construct | **Operationalisation** |
| Recruitment | Which routes of access were utilized? |
| Involvement | Were techniques for involvement described? Which measures were taken to enable the involvement of people with dementia? |
| Research question 1b: Which forms of involvement enable people with dementia to participate actively throughout the entire research process? | |
| Forms of involvement | Which forms of involvement of people with dementia were described? |
| Involvement across the research process | In which phases of the research process was the involvement of people with dementia described? How was involvement across the research process evaluated and assessed? |

**Operationalisation of research question 2 (part 2, qualitative interview study)**

**Aim (2)**: To explore experiences of collaboration within the PPIE group of the eSENIORS project, from the perspective of PwD and other involved stakeholders, and to identify facilitating and hindering factors for subjectively beneficial PPIE practice.

Research question 2a: From the perspective of PPIE group participants, which factors facilitate beneficial collaboration with researchers?

Research question 2b: From the perspective of PPIE group participants, which factors hinder collaboration with researchers?

Research question 2c: How do members of the PPIE group evaluate the way researchers handled their contributions and perspectives?

| Standard (NIHR) | Operationalised aspect | Interview guide |
| --- | --- | --- |
| Inclusive Opportunities | Was participation designed to be easily accessible? What barriers or facilitators were there for joining and taking part? | Initial experiences of joining; reasons for staying involved; hindering and facilitating factors for participation |
| Working together | How was collaboration within the group experienced? Was there a sense of group cohesion? | Sense of group belonging; collaboration within the group; expectations of the group |
| Support and learning | Was support offered? Were there any orientation opportunities? How was support experienced or desired? | Opportunities to ask questions; questions about specific information that participants felt was missing |
| Governance | Was there transparency regarding roles, responsibilities and decisions? How accessible was decision-making? | Freedom to contribute to shaping the work; perceived influence of contributions |
| Communication | How was information communicated? Were the language and channels understandable and accessible? | Exchange with researchers; forms of communication; feedback |
| Impact | How were contributions handled? Was any effect or influence perceived? | Handling of input; perceived impact; feedback culture |

**Topic guide**

**Participants**: Members of the eSENIORS PPIE group

**Duration**: max. 60 minutes

1. Introduction / for participating people with dementia, more time will be allocated
2. Verbal re-consent / for participating people with dementia, more time will be allocated and, where appropriate, verbal re-consent will also be checked during the interview
3. Clarification of questions regarding the interview or the study / for participating people with dementia, more time will be allocated

- **Start audio recorder!**

Information: This interview is intended to collect feedback from the group in order to identify facilitating and hindering factors in the collaboration. We would also like to understand how the group evaluates the way its input was handled.

4. Topics to be explored

| **Topic** | **Opening invitation** | **Memos for follow-up questions** | **Prompts / follow-up questions / steering if content is sparse** |
| --- | --- | --- | --- |
| Joining the group | To begin with, could you please tell me what motivated you to become a member of this group? | Ask about initial experiences of joining. Expectations of oneself and of the group. Reasons for continuing participation. | At the last workshop, you took part in the PPI group for the first time. How did that feel for you? What expectations do you have of yourself and of the group? |
| Participation | When you think back to the meetings, what was it like for you to come together with the other participants? | Group experience / group cohesion. Facilitating and hindering factors. Freedom to help shape the process. | How would you describe the collaboration within the group? How did you find your place in the group? What made it easier for you to contribute to the conversation at the table? What made it more difficult for you to contribute? → probe directly, for example: “Would you have liked to say more...?” |
| Communication and exchange with researchers | How did you experience the exchange and conversations with the researchers before, during and after the meetings? | Comprehensibility, design (e.g., tasks/assignments). Forms of communication (email, telephone, etc.). | Would you have preferred other forms of communication beforehand (e.g., a phone call)? Do you have suggestions for how tasks/assignments could be designed better? |
| Handling of input | From your point of view, how was what you and the group contributed during the discussions at the table dealt with? | Perceived impact, appreciation, feeling heard. Feedback from the research team. | What showed you that your contributions were taken seriously? How do you evaluate the feedback or information you received from the research team? Are there any specific pieces of information that you feel were missing? |
| Evaluation | Looking back at the collaboration in the last workshop: – What did you particularly like? – What would you change? | Points of criticism, wishes for change, ideas for improvement. | Was there anything… that you particularly liked or missed, or that you would have wished for or imagined differently? |

5. Thank participants, closing
