# Supplementary material for: Involving People With Dementia in Research: Contributions, Barriers and Evaluation From a Meta‐Synthesis and Qualitative Interview Study
Source: Health Expect. 2026 Sep 7;29(5):e70864. doi: 10.1111/hex.70864 (PMC13548409; doi:10.1111/hex.70864)
Supplement: Supplementary file 4 [file HEX-29-e70864-s004.docx]

**Date of Search:** 05.09.2025

**PubMed:**

| **#** | **Search (05.09.2025)** | **Results** |
| --- | --- | --- |
| 1 | "Dementia"[MeSH Terms] OR "Alzheimer Disease"[MeSH Terms] OR "Memory Disorders"[MeSH Terms] OR "Cognitive Dysfunction"[MeSH Terms] | 284’122 |
| 2 | "Dementia"[Title/Abstract] OR "cognitive dysfunction"[Title/Abstract] OR "mild cognitive impairment"[Title/Abstract] OR "alzheimer disease"[Title/Abstract] OR "memory disorders"[Title/Abstract] OR "MCI"[Title/Abstract] OR "AD"[Title/Abstract] | 378’744 |
| 3 | "Community-Based Participatory Research"[MeSH Terms] OR "Citizen Science"[MeSH Terms] | 6,904 |
| 4 | "Patient and public Involvement"[Title/Abstract] OR "service user involvement"[Title/Abstract] OR "Patient and public involvement and engagement"[Title/Abstract] OR "patient participation"[Title/Abstract] OR "Patient and public voice"[Title/Abstract] OR "study partner"[Title/Abstract] OR "participatory research"[Title/Abstract] OR "consumer participation"[Title/Abstract] OR "collaborative research"[Title/Abstract] OR "Co-research"[Title/Abstract] OR "citizen participation"[Title/Abstract] OR "PPIE"[Title/Abstract] | 20’062 |
| 5 | "recruit*"[Title/Abstract] OR "engag*"[Title/Abstract] OR "inclu*"[Title/Abstract] OR "involv*"[Title/Abstract] OR "participatory method*"[Title/Abstract] OR "participation"[Title/Abstract] OR "empower*"[Title/Abstract] | 9'106’287 |
| 6 | ((#1 OR #2) AND (#3 OR #4)) AND (#5) | 508 |
| 7 | (((“semi-structured”[TIAB] OR semistructured[TIAB] OR unstructured[TIAB] OR informal[TIAB] OR “in-depth”[TIAB] OR indepth[TIAB] OR “face-to-face”[TIAB] OR structured[TIAB] OR guide[TIAB] OR guides[TIAB]) AND (interview*[TIAB] OR discussion*[TIAB] OR questionnaire*[TIAB])) OR (“focus group”[TIAB] OR “focus groups”[TIAB] OR qualitative[TIAB] OR ethnograph*[TIAB] OR fieldwork[TIAB] OR “field work”[TIAB] OR “key informant”[TIAB])) OR “interviews as topic”[Mesh] OR “focus groups”[Mesh] OR narration[Mesh] OR qualitative research[Mesh] OR "personal narratives as topic"[Mesh] OR (theme[TIAB] OR thematic[TIAB]) OR "ethnological research"[TIAB] OR phenomenol*[TIAB] OR "grounded theory"[TIAB] OR "grounded study"[TIAB] OR "grounded studies"[TIAB] OR "grounded research"[TIAB] OR "grounded analysis"[TIAB] OR "grounded analyses"[TIAB] OR "life story"[TIAB] OR "life stories"[TIAB] OR emic[TIAB] OR etic[TIAB] OR hermeneutics[TIAB] OR heuristic*[TIAB] OR semiotic[TIAB] OR "data saturation"[TIAB] OR "participant observation"[TIAB] OR "action research"[TIAB] OR "cooperative inquiry"[TIAB] OR "co-operative inquiry"[TIAB] OR "field study"[TIAB] OR "field studies"[TIAB] OR "field research"[TIAB] OR "theoretical sample"[TIAB] OR "theoretical samples"[TIAB] OR "theoretical sampling"[TIAB] OR "purposive sampling"[TIAB] OR "purposive sample"[TIAB] OR "purposive samples"[TIAB] OR "lived experience"[TIAB] OR "lived experiences"[TIAB] OR "purposive sampling"[TIAB] OR "content analysis"[TIAB] OR discourse[TIAB] OR "narrative analysis"[TIAB] OR heidegger*[TIAB] OR colaizzi[TIAB] OR spiegelberg[TIAB] OR "van manen*"[TIAB] OR "van kaam"[TIAB] OR "merleau ponty"[TIAB] OR husserl*[TIAB] OR Foucault[TIAB] or Corbin[TIAB] OR Strauss[TIAB] OR Glaser[TIAB] | 809’584 |
| 8 | **(((#1 OR #2) AND (#3 OR #4)) AND (#5)) AND (#7)** | 224 |

Seach #6 was then translated using the Polyglot search translator from the Systematic Research Accelerator (sr-accelerator.com). This resulted in search strings for CINAHL PsycINFO, which were then completed with their specific filters. The result are the following searches:

**CINAHL:**

| **#** | **Search (05.09.2025)** | **Results** |
| --- | --- | --- |
| 1 | ((MH Dementia+) OR (MH "Alzheimer Disease+") OR (MH "Memory Disorders+") OR (MH "Cognitive Dysfunction+") OR ((TI Dementia OR AB Dementia) OR (TI "Cognitive Dysfunction" OR AB "Cognitive Dysfunction") OR (TI "mild cognitive impairment" OR AB "mild cognitive impairment") OR (TI "Alzheimer Disease" OR AB "Alzheimer Disease") OR (TI "Memory Disorders" OR AB "Memory Disorders") OR (TI MCI OR AB MCI) OR (TI AD OR AB AD))) AND ((MH "Community-Based Participatory Research+") OR (MH "Citizen Science+") OR ((TI "Patient and public Involvement" OR AB "Patient and public Involvement") OR (TI "service user involvement" OR AB "service user involvement") OR (TI "Patient and public involvement and engagement" OR AB "Patient and public involvement and engagement") OR (TI "patient participation" OR AB "patient participation") OR (TI "Patient and public voice" OR AB "Patient and public voice") OR (TI "study partner" OR AB "study partner") OR (TI "participatory research" OR AB "participatory research") OR (TI "consumer participation" OR AB "consumer participation") OR (TI "collaborative research" OR AB "collaborative research") OR (TI Co-research OR AB Co-research) OR (TI "citizen participation" OR AB "citizen participation") OR (TI PPIE OR AB PPIE))) AND ((TI recruit* OR AB recruit*) OR (TI engag* OR AB engag*) OR (TI inclu* OR AB inclu*) OR (TI involv* OR AB involv*) OR (TI "participatory method*" OR AB "participatory method*") OR (TI participation OR AB participation) OR (TI empower* OR AB empower*)) | 199 |
| 2 | AND (MH “action research”) or (MH "Audiorecording") or (MH "cluster sample+") or (MH "constant comparative method") or (MH "content analysis") or (MH "discourse analysis") or (MH "ethnographic research") or (MH "ethnological research") or (MH "ethnography") or (MH "ethnonursing research") or (MH "field studies") or (MH "focus groups") or (MH "grounded theory") or (MH "Historical Records") or (MH "Interviews+") or (MH "Narratives") or (MH “naturalistic inquiry”) or (MH "observational methods+") or (MH "phenomenological research") or (MH "phenomenology") or (MH "purposive sample") or (MH "qualitative studies") or (MH "qualitative validity+") or (MH "questionnaires") or (MH "thematic analysis") or (MH "theoretical sample") or (MH "Videorecording+") or TX colaizzi* or TX constant comparative or TX constant comparison or TX cooperative inquir* or TX co-operative inquir* or TX co operative inquir* or TX Corbin* TX data saturat* or TX discourse* analysis or TX emic or TX etic or TX ethnon* or TX field research or TX field stud* or TX focus group* or TX Foucault* or TX giorgi* or TX Glaser* or TX grounded analysis or TX grounded research or TX grounded studies or TX grounded study or TX grounded theor* or TX heidegger* or TX hermeneutic* or TX heuristic or TX human science or TX husserl* or TX life experiences or TX life stor* or TX lived experience* or TX merleau ponty* or TX narrative analysis or TX qualitative or TX participant observ* or TX phenomenol* or TX purpos* sampl* or TX questionnaire* or TX semiotics or TX spiegelberg* or TX Strauss* TX van kaam* or TX van manen* | 1'770’715 |
| 3 | #1 AND #2 | 94 |

**PsycINFO**

| **#** | **Search (05.09.2025)** | **Results** |
| --- | --- | --- |
| 1 | (exp Dementia/ OR exp "Alzheimer Disease"/ OR exp "Memory Disorders"/ OR exp "Cognitive Dysfunction"/ OR (Dementia.ti,ab. OR "Cognitive Dysfunction".ti,ab. OR "mild cognitive impairment".ti,ab. OR "Alzheimer Disease".ti,ab. OR "Memory Disorders".ti,ab. OR MCI.ti,ab. OR AD.ti,ab.)) AND (exp "Community-Based Participatory Research"/ OR exp "Citizen Science"/ OR ("Patient and public Involvement".ti,ab. OR "service user involvement".ti,ab. OR "Patient and public involvement and engagement".ti,ab. OR "patient participation".ti,ab. OR "Patient and public voice".ti,ab. OR "study partner".ti,ab. OR "participatory research".ti,ab. OR "consumer participation".ti,ab. OR "collaborative research".ti,ab. OR Co-research.ti,ab. OR "citizen participation".ti,ab. OR PPIE.ti,ab.)) AND (recruit*.ti,ab. OR engag*.ti,ab. OR inclu*.ti,ab. OR involv*.ti,ab. OR "participatory method*".ti,ab. OR participation.ti,ab. OR empower*.ti,ab.) | 199 |
| 2 | (experiences.tw. OR interview:.tw. OR qualitative.tw.) | 826’730 |
| 3 | #1 AND #2 | 84 |

**LILACS**

For LILACS, the search string was translated into Spanish using ChatGPT. This yielded the following search:

| **#** | **Search (05.09.2025)** | **Results** |
| --- | --- | --- |
| 1 | ((demência OR "doença de Alzheimer" OR "transtornos de memória" OR "disfunção cognitiva" OR "mild cognitive impairment" OR MCI OR AD) AND ("participação do paciente" OR "envolvimento do paciente" OR "pesquisa participativa" OR "pesquisa colaborativa" OR "ciência cidadã" OR "co-pesquisa" OR "iniciativa do cidadão" OR "patient and public involvement" OR PPIE) AND (recrut* OR envolv* OR inclu* OR particip* OR engaj* OR empoder*)) AND (qualitativa OR "pesquisa qualitativa" OR "análise de conteúdo" OR "grupos focais" OR "entrevistas" OR "teoria fundamentada" OR fenomenologia OR "análise temática" OR observação OR narrativas OR "amostragem intencional") | 6 |

**Search prompts for the AI-tool Elicit (05.09.2025):**

1) What are procedures and techniques that can support the recruitment and involvement of people with dementia in Patient and public involvement groups?

2) What are forms of participation that enable people with dementia to actively contribute throughout the entire research process in patient and public involvement?

**Non-duplicate results: 5**
